# Supplementary material for: Single Stem Cell Imaging and Analysis Reveals Telomere Length Differences in Diseased Human and Mouse Skeletal Muscles
Source: Stem Cell Reports. 2017 Sep 7;9(4):1328–41. doi: 10.1016/j.stemcr.2017.08.003 (PMC5639167; doi:10.1016/j.stemcr.2017.08.003)
Supplement: Document S1. Supplemental Experimental Procedures, Figures S1–S7, and Tables S1–S5 [file mmc1.pdf]

Stem Cell Reports, Volume *9*

Supplemental Information

Single Stem Cell Imaging and Analysis Reveals Telomere Length Differences in Diseased Human and Mouse Skeletal Muscles

Elisia D. Tichy, David K. Sidibe, Matthew T. Tierney, Michael J. Stec, Maryam Sharifi-Sanjani, Harish Hosalkar, Scott Mubarak, F. Brad Johnson, Alessandra Sacco, and Foteini Mourkioti

Figure S1

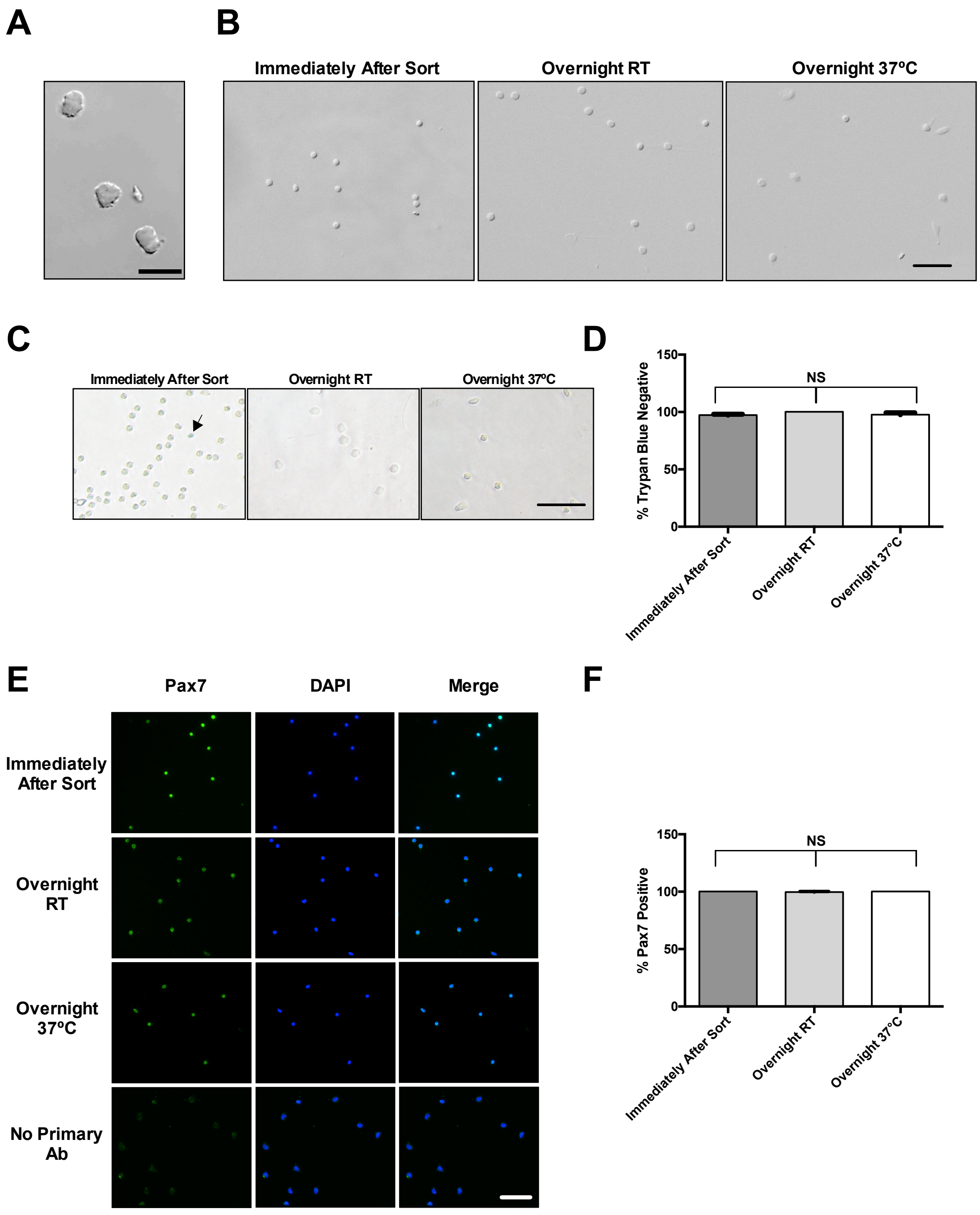

Figure S1. Telomere MuQ-FISH Processing Does Not Affect MuSC Appearance, Cell Death, or Differentiation Status. Related to Figure 1.

A) Representative Differential Interference Contrast (DIC) image of FACS-isolated mMuSCs plated on laminin-coated plates. Scale bar: 10 µm.

B) Murine MuSCs were plated on laminin-coated chamber slides and allowed to attach for 30 minutes before fixation (immediately after sort), plated and cultured overnight at 37ºC before fixation, or plated overnight at room temperature and dried before fixation. Cells were imaged by DIC microscopy. Scale bar: 100 µm.

C) MuSCs were plated as described in (B) and trypan blue was added to the culture media. In the case of the room temperature overnight group, trypan blue was added with media, to create the same final concentration. Cells were imaged by phase contrast microscopy. Scale bar: 50 µm.

D) Analysis of the percent of live cells (trypan blue negative), based on images taken in (C). n=7 mice analyzed. N>100 cells analyzed per condition. Displayed is mean ± SEM.

E) MuSCs were plated as in (B), fixed, and stained for Pax7, an undifferentiated MuSC marker. Scale bar: 100 µm.

F) Quantitation of data from (E). n=7 mice analyzed. N>50 cells analyzed per condition. Displayed is mean ± SEM.

Figure S2

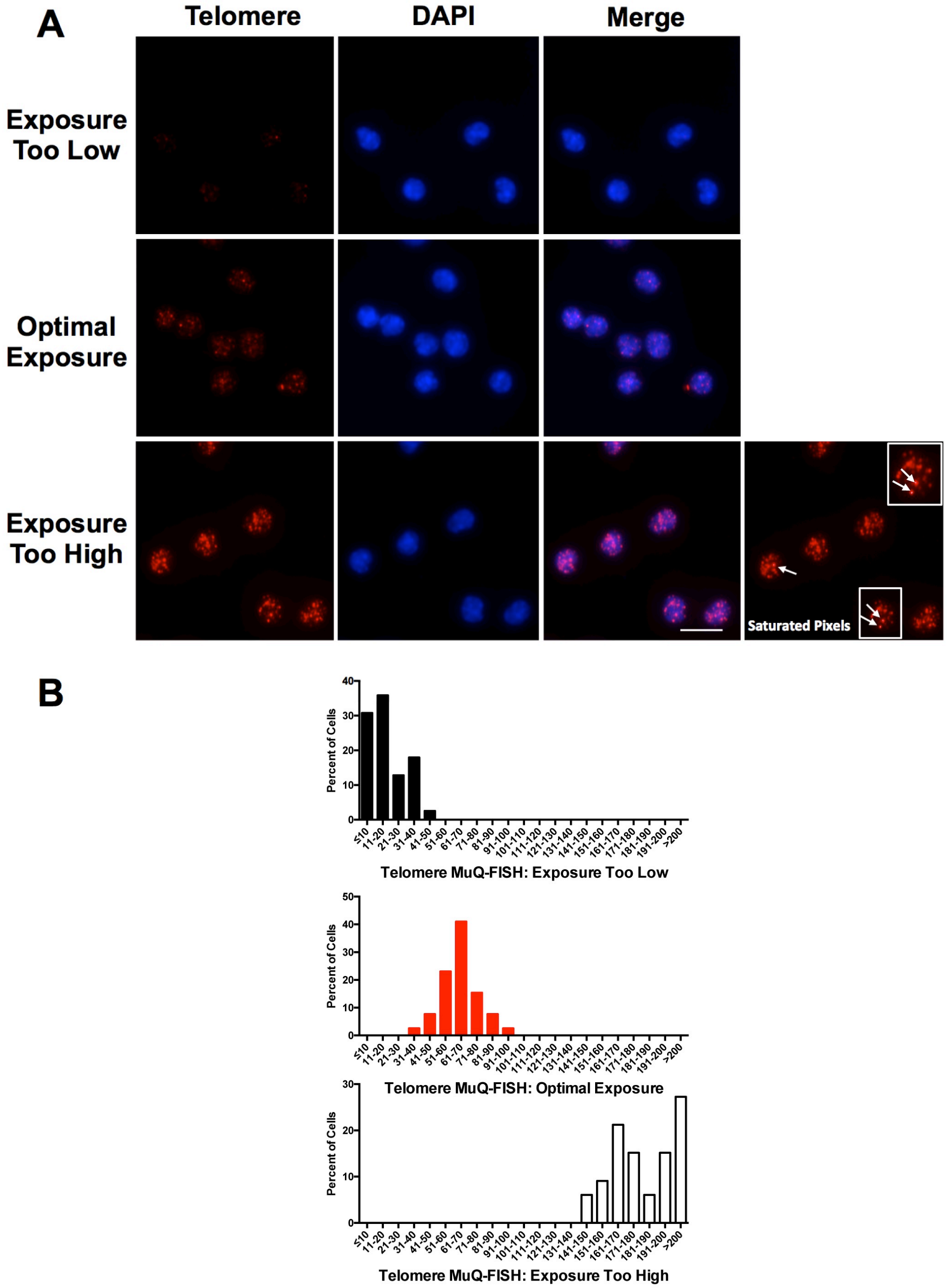

Figure S2. Determination of Optimal Exposures for MuQ-FISH. Related to Figure 1.

A) WT mMuSCs were processed for Telomere MuQ-FISH and imaged at different exposures for Cy3. It is necessary to image experimental groups to determine optimal exposure settings before collecting data. In this case, the exposure too low settings for Cy3 were 200 ms; 150 visual gain. For the optimal setting for this experiment, Cy3 was imaged at 300 ms; 150 visual gain. For the exposure too high setting where saturated

pixels can be observed (white arrows), exposure settings for Cy3 were 800 ms; 150 visual gain. DAPI images were taken at the same settings for all groups (50 ms; 206 visual gain for this experiment). Oversaturated pixels were determined using Nikon elements software. Scale bar: 5 µm. Exposure settings will vary from lots of reagents and the age of the mercury bulb in the microscopy equipment.

B) MuQ-FISH was conducted on WT MuSCs imaged with different Cy3 intensities. Data are displayed as histograms using the sum intensity information. n>30 cells/group analyzed.

Figure S3

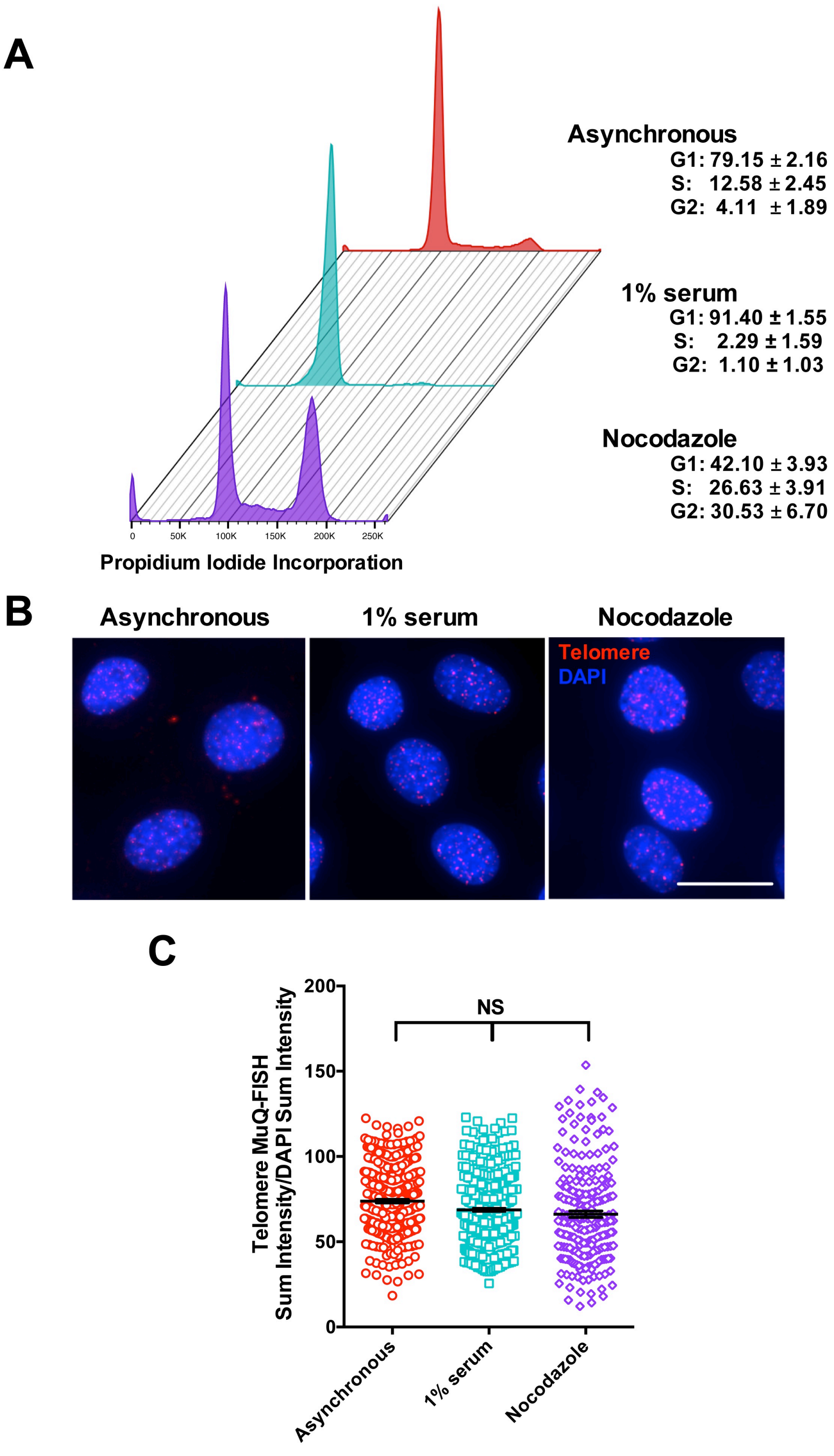

Figure S3. Cell Cycle Phase Does Not Affect Telomere Length Analysis by Telometer Related to Figure 1.

A) Undifferentiated C2C12 cells were enriched in G1 or G2 phases of the cell cycle or allowed to grow asynchronously. Cells were harvested and analyzed for cell cycle position by flow cytometry. Results were calculated from n=3 biological replicates.
B) Representative images of cells described in (A) that were processed and stained for MuQ-FISH. Cells with condensed chromatin (mitotic) were not included in the analysis. Scale bar: 25 µm.
C) Analysis of C2C12 cells by MuQ-FISH. Sum intensity method is displayed. Data presented as mean ± SEM. At least 250 cells were analyzed per group. Displayed is mean ± SEM.

Figure S4

A

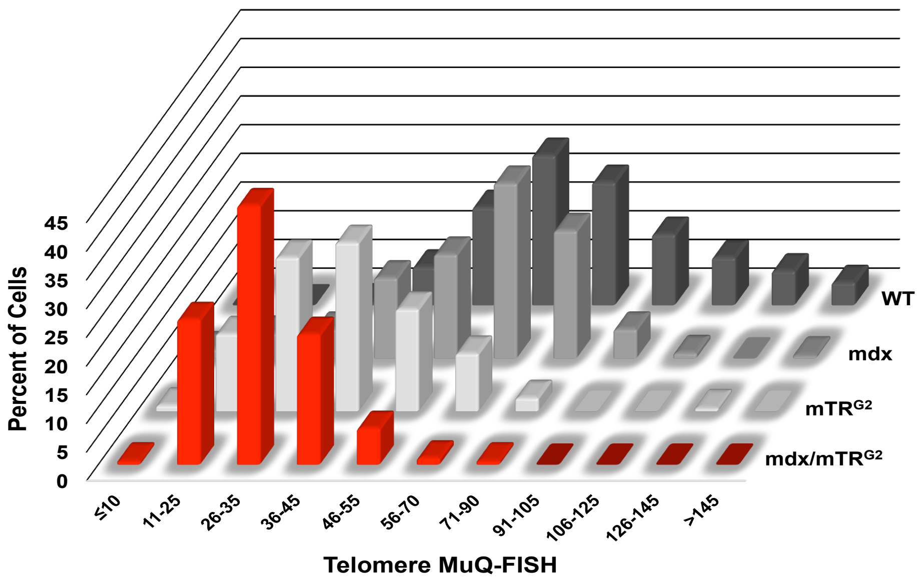

B

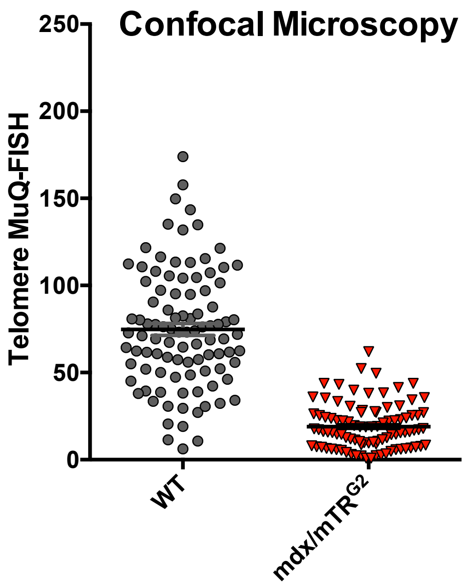

Figure S4. Telomere Shortening in Dystrophic mMuSCs. Related to Figure 4.

A) Alternative representation of the histogram in presented in Figure 4C.

B) Confocal analysis of MuQ-FISH. MuSCs from 4 WT or 4 mdx/mTR^{G2} mice were plated and processed for MuQ-FISH. Cells were imaged on a Zeiss LSM 710 confocal microscope, with 5-8 sections of 1µm per slice were taken per image. Slices were

combined in Fiji using the Z-project maximum projection algorithm. Combined Z-images of DAPI and of Cy3 were analyzed by Telometer. N>90 cells analyzed per genotype.

Figure S5

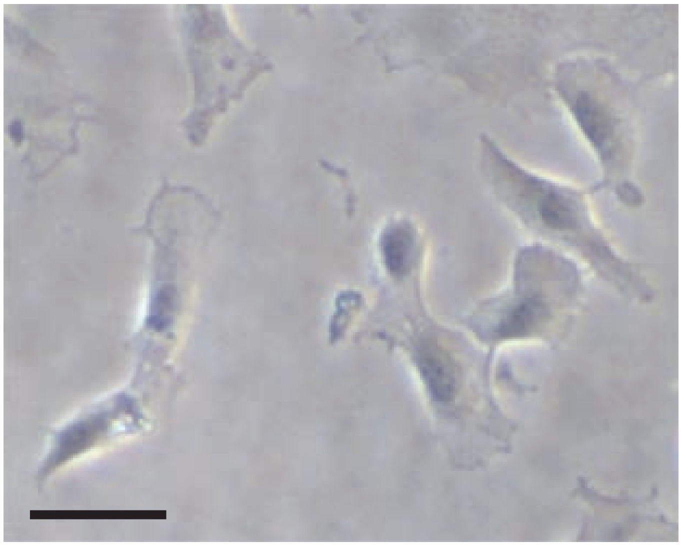

Figure S5. Morphology of Cultured hMuSCs. Related to Figure 5.
A representative image of hMuSC cells grown on collagen-coated slides, fixed, and imaged by phase contrast microscopy. Scale bar: 50 μm

Figure S6

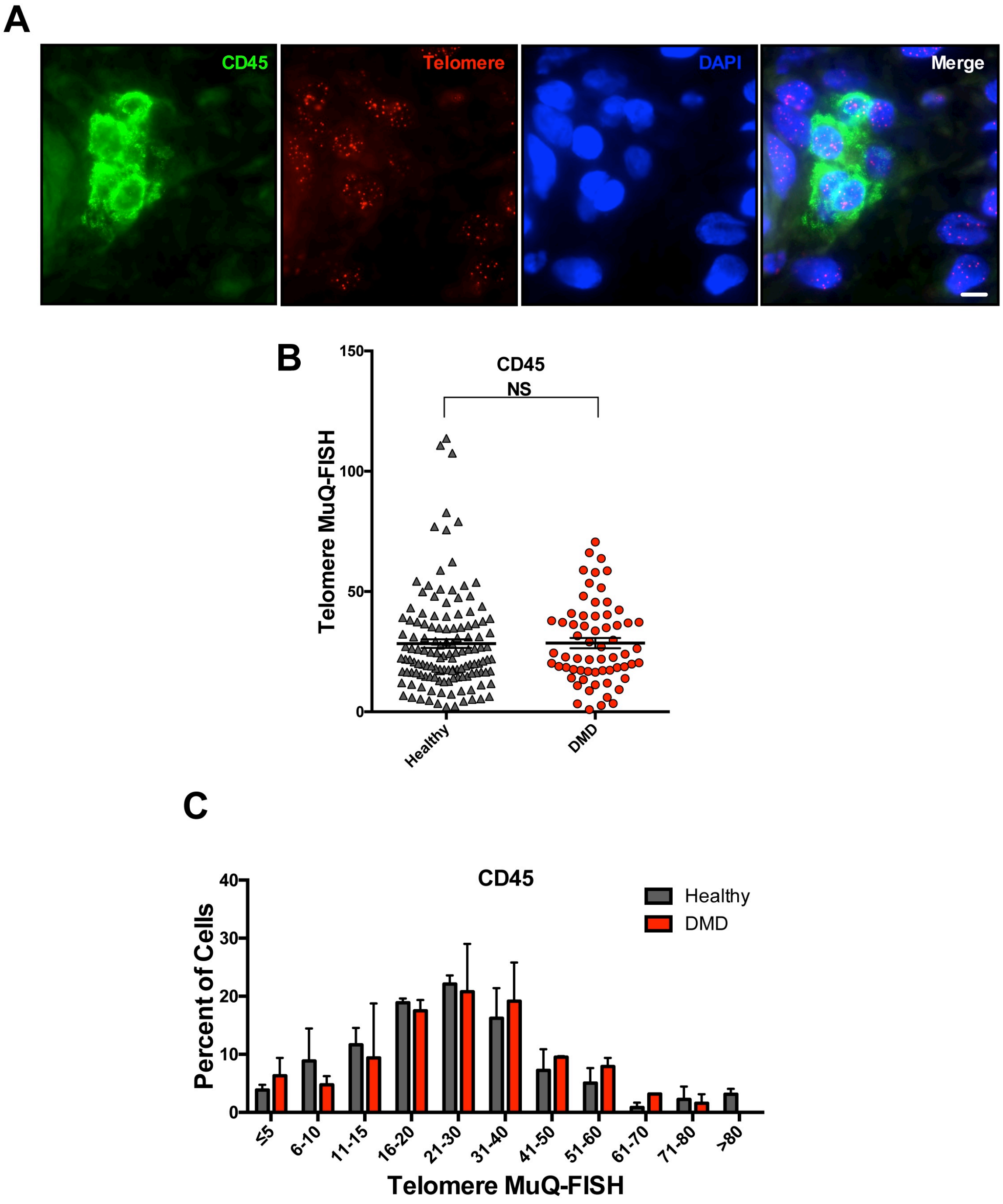

Figure S6. MuQ-FISH Staining of Leukocytes in Human Patient Samples. Related to Figure 6.

A) Skeletal muscle cryosections from human healthy or DMD-diseased patients were processed for MuQ-FISH and stained with the leukocyte marker CD45 (green). Scale bar: 10 μm.

B) Telomere length was assessed by MuQ-FISH (sum intensity) in $CD45^+$ cells in human healthy or DMD-diseased cryosections. N=3-4 patient samples per condition, n>60 $CD45^+$ cells analyzed per group. Displayed is mean ± SEM.

C) Histogram depiction of data shown in B. Displayed is mean ± SEM.

Figure S7

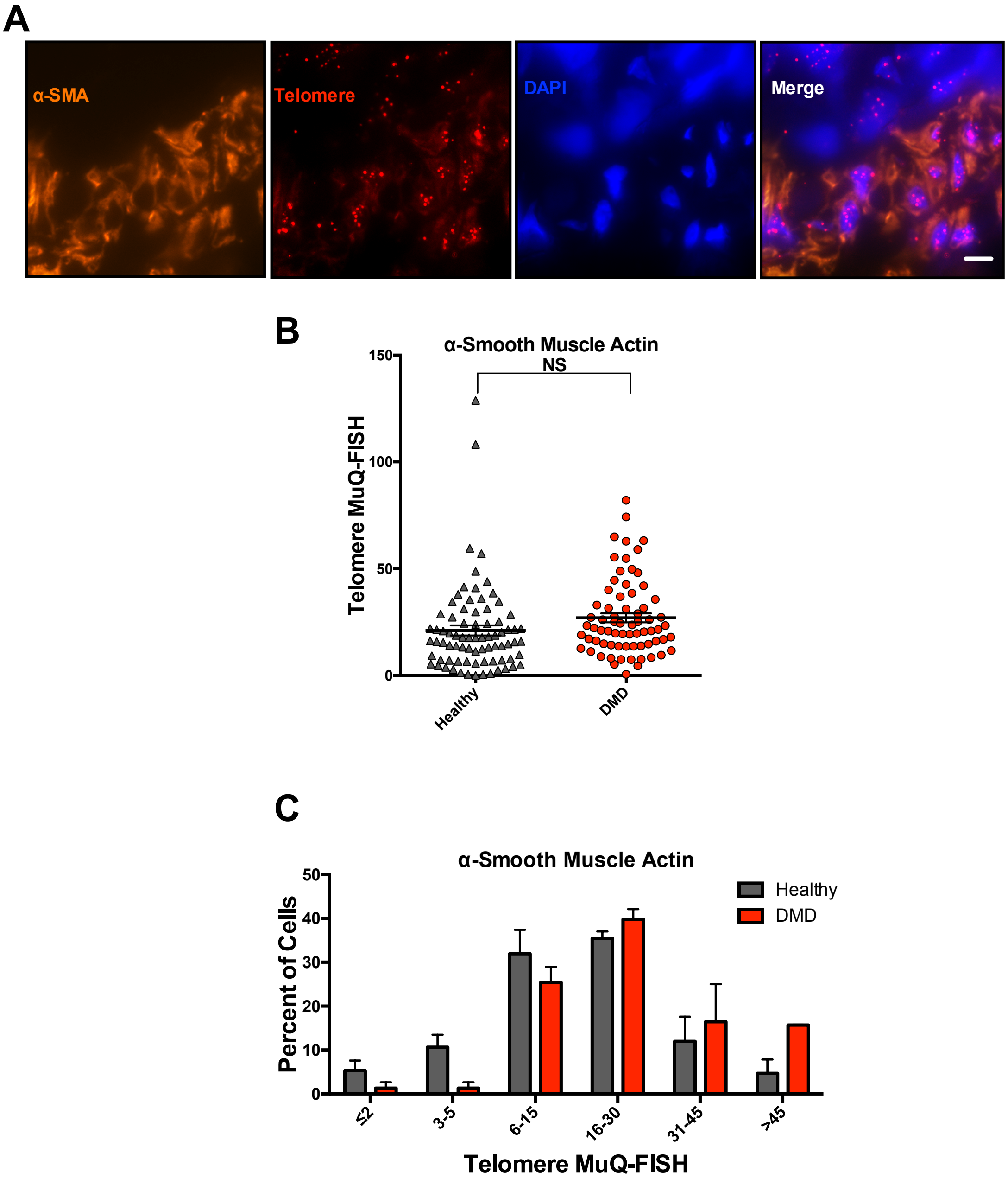

Figure S7. MuQ-FISH Staining of Smooth Muscle Cells in Human Patient Samples Related to Figure 6.

A) Skeletal muscle cryosections from human healthy or DMD-diseased patients were processed for MuQ-FISH stained with alpha smooth muscle actin (orange). Scale bar: 10 µm.

B) Telomere length was assessed by MuQ-FISH (sum intensity) in a α-SMA$^+$ cells in human healthy or DMD-diseased cryosections. N=3-4 patient samples per condition, n=70 α-SMA$^+$ cells analyzed per group. Displayed is mean ± SEM.

C) Histogram depiction of data shown in B. Displayed is mean ± SEM

Table S1. Antibodies and Staining Reagents Used in mMuSC FACS. Related to Figures 1-4.

Antigen	Host	Clone	Conjugate	Source	Dilution
**CD45**	Rat	30-F11	Biotin	BD Biosciences	1/500
**CD11b**	Rat	M1/70	Biotin	BD Biosciences	1/200
**CD31**	Rat	390	Biotin	eBioscience	1/200
**Sca1/Ly6A/E**	Rat	E13-161.7	Biotin	BD Biosciences	1/200
**Live/Dead**	N/A	N/A	7-aminoactinomycin D (7-AAD) or DAPI	Sigma-Aldrich/Thermo Fisher	1/250
**Streptavidin**	N/A	N/A	PE-Cy7 or APC-Cy7	Biolegend or BD Biosciences	1/25
**Alpha 7-Integrin**	Rat	R2F2	AF 647 or AF 488	Ablab	1/25
**CD34**	Rat	Ram34	BV421 AF 647	BD Biosciences	1/12.5

Table S2. Antibodies and Staining Reagents for hMUSC isolation. Related to Figure 5 and 6.

Antigen/Reagent	Host	Clone	Conjugate	Source	Dilution
**CD45**	Mouse	HI30	Biotin	eBioscience	1/250
**CD11b**	Mouse	ICRF44	Biotin	eBioscience	1/100
**CD31**	Mouse	WM59	Biotin	eBioscience	1/100
**Propidium Iodide**	N/A	N/A	N/A	Thermo Scientific	1/1000
**Streptavidin**	N/A	N/A	APC-Cy7	BD Biosciences	1/100
**NCAM/CD56**	Mouse	CMSSB	APC	eBioscience	1/100

Table S3: Human Subject Information. Related to Figure 5 and 6.

Patient	Group	Age (y)	Muscle	MuSC Yield
1	Healthy	13.7	Vastus lateralis	12,200
2	Healthy	14.7	Vastus lateralis	6,450
3	Healthy	14.2	Vastus lateralis	5,600
4	DMD	13.8	Gastrocnemius/soleus	2,700
5	DMD	11.0	Gastrocnemius/soleus	1,000
6	DMD	10.3	Gastrocnemius	3,400

Table S4. Fluorophores and Aria Filter Sets. Related to Figures 1-6.

Fluor	Laser	Filter
**7-AAD**	Blue	710/50
**Alexa Fluor 488**	Blue	530/30
**Alexa Fluor 647**	Red	660/20
**APC**	Red	660/20
**APC-Cy7**	Red	780/60
**BV421**	Violet	450/50
**DAPI**	Violet	450/50
**Propidium Iodide**	Blue	585/42
**PE-Cy7**	Green	780/60

Table S5. Formulas Used for MuQ-FISH Analysis. Related to Figures 2, 3, 4, 6.

**Sum Intensity**	$\frac{\textbf{Sum Intensity of Telomere Signal}}{\textbf{Sum Intensity of DAPI Signal}}$
**Mean Intensity**	$\frac{\textbf{Mean Intensity of Telomere Signal}}{\textbf{Sum Intensity of DAPI Signal}}$

Note: After ratio calculations, all values were multiplied by a conversion factor to generate values greater than 1.

Extended Experimental Procedures

Mice

Wildtype C57Bl/6J mice were purchased from Jackson Labs (stock #000664). Other mice used in the study included mdx (Jackson Labs stock #002378), mTR^{G1} (generation 1 mTR knockout, Jackson Labs stock #004132, derived from a heterozygous cross) mTR^{G2} (generation 2; generated through in-house by breeding homozygous Jackson Labs stock #004132), mTR^{G3} (generation 3) and mdx/mTR^{G2} (generation 2), the latter of which were generated through an in-house breeding, as described previously (Mourkioti et al., 2013; Sacco et al., 2010).

Murine Muscle Digestion and Cell Staining

Mice were sacrificed, and the tibialis anterior, quadriceps and gastrocnemius muscles were dissected from both hind legs. Muscle was finely minced and placed in a gentleMACS C tube (Miltenyi Biotec) containing 0.15% collagenase in 10 mL DMEM. Tubes were loaded into a MACS Dissociator (Miltenyi Biotec) and the manufacturer's spleen-02 program was run twice. Tubes were incubated in a humidified 37ºC/5% CO_2 incubator for 30 min, subjected to the spleen-02 program again, and incubated at 37ºC for 1 hr. Seventy five microliters of 2% collagenase (Sigma-Aldrich) and 75 µL of 4.8 U/mL dispase (Roche) was added, and tubes were vortexed at maximum speed prior to a 30-min incubation at 37ºC. Cells were passed through a 21-gauge needle until all muscle was broken apart. The remaining cell slurry was filtered through a 40 µM cell strainer that was prewet with 10 mL of cold myoblast media [DMEM:F12; 15% FBS, 1X anti-anti (Amphotericin B, Penicillin, Streptomycin); all from Gibco]; the strainer was rinsed with an additional 10 mL of cold myoblast media, and cells were pelleted at 300x*g*/4ºC. Cells were incubated with 1mL 1X red cell lysis buffer (eBioscience) for 5 min at room temperature and 9 mL cold FACS buffer (2.5% goat serum, 2 mM EDTA, pH 8.0 in 1X PBS) was added. Cells were spun and resuspended in 1mL of FACS buffer containing antibodies raised against antigens CD45, CD31, CD11b, and Sca1 (see Table S1) prior to an incubation on ice for 45 min. Cells were centrifuged and resuspended in 100 µL of FACS buffer containing antibodies CD34, and α7-integrin, as well as streptavidin-PE-Cy7 (Table S1). Cells were incubated in the dark for 90 min on ice, with agitation occurring every 30 min. FACS buffer was added up to 1 mL final volume, and cells pelleted and resuspended in FACS buffer, and the viability dye 7-aminoactinomycin D (7-AAD; Sigma-Aldrich) was added (final concentration 4 µg/mL). Cells were placed in flow cytometry tubes with cell strainers (BD Biosciences) before collection by FACS. In some cases, the antibody fluorescent conjugate choice was altered (see Table S1).

Human Muscle Biopsy Digestion and Cell Staining

Human biopsies were enzymatically dissociated (0.2% collagenase and 0.02 units/mL dispase, Sigma-Aldrich) for 45 min at 37ºC, minced under a dissection microscope and incubated for an additional 45 min. The resulting cell suspension was filtered through a 70 µm nylon filter and incubated with the following biotinylated antibodies: CD45, CD11b and CD31 (eBioscience; See Table S2). Samples were washed and incubated with anti-NCAM/CD56 (eBioscience) and Streptavidin APC-Cy7 (BD Biosciences). Dead cells were excluded by Propidium Iodide incorporation (Thermo Scientific) before fractionation of the $CD45^{-}CD11b^{-}CD31^{-}NCAM/CD56^{+}$ population by flow cytometry (BD FACS Aria II). Purity checks were performed by re-sorting an aliquot of each sorted population. Patient sample metrics can be found in Table S3.

Fluorescence-Activated Cell Sorting (FACS)

Both mMuSCs and hMuSCs were sorted using a BD Aria II. Information about lasers and filter sets can be found in Table S4. mMuSCs were sorted into 1.5 mL microcentrifuge tubes containing 500 µL of cold myoblast media [DMEM/Ham's F12; 15% FBS; 1X anti-anti (Fisher Scientific)]. The collection tubes were maintained at 4ºC during the sort process using a circulating water system. Data was collected from 20,000 total events, unless otherwise noted, and analyzed using FlowJo 10.1 software.

hMuSC culture

hMuSCs were plated on tissue culture plates coated with collagen (BD Biosciences) and maintained in growth media composed of Ham's F-10 (Gibco) or DMEM/Ham's F12 (Gibco) and 15% FBS (Omega Scientific). hMuSCs were expanded and passaged by dissociation with Accumax (Millipore). Healthy lines were used at passages 7-10 for this study, whereas DMD lines were used at passages 5-7.

Trypan Blue Staining

To determine viability, MuSCs were plated in laminin-coated 16 well chamber slides in 100 µL myoblast media. Cells were allowed to attach for 30 minutes in a 37ºC/5% CO_2 incubator. Trypan blue (Corning; 1/10 dilution of a 0.4% w/v solution in PBS) was added to the media and cells were imaged by phase microscopy (immediately after sort). MuSCs were also plated on chamber slides and left overnight in the incubator or placed at room temperature without the lid to dry. The following day, trypan blue was added to the culture media for cells in the incubator, or mixed up 1/10 with myoblast media and added to the dried room temperature wells. Cells were also imaged by phase microscopy.

Pax7 Staining

Mouse MuSCs were plated on laminin-coated 4-well chamber slides in a manner identical to the trypan blue staining procedure above. Cells were fixed with 4% paraformaldehyde/PBS for 20 min and washed once with PBS. Cells were permeabilized with 0.5% triton X-100/PBS for 30 min, washed twice with PBS, and blocked for 1 hr at room temperature in 3% BSA/PBS. Cells were stained for Pax7 (santa cruz; clone PAX7; 1/50 in 3% BSA/PBS) overnight at 4ºC, washed with PBS, and stained with Alexa Fluor 488-conjugated goat anti-mouse IgG (Life Technologies; 1/500 in 3% BSA/PBS) at room temperature for 1 hr. Cells were washed, chambers removed, and coverslips were mounted with fluoromount G plus DAPI (SouthernBiotech).

Supplemental Imaging of mMuSCs and hMuSCs.

Isolated mMuSCs were plated on laminin-coated slides. hMuSCs were grown on collagen-coated chamber slides. Both cell types were fixed with 4% paraformaldehyde/PBS for 10 min, and coverslips were mounted with Fluoromount G. mMuSCs were imaged by differential interference contrast microscopy using a Leica SP8 confocal microscope with a Nikon Plan Apo CS2 63x/1.40 oil objective. hMuSCs were imaged with a Nikon TE300 inverted CCD SPOT RT camera using a 10X air objective. For DIC imaging of mMuSCs plated and harvested at different time points after sorting, mMuSCs were plated as described in trypan blue staining above, fixed in 4% paraformaldehyde in PBS, and coverslips were mounted with fluoromount G (SouthernBiotech) before imaging.

Human Muscle Cryosection MuQ-FISH

Cryosections (10 µm slices) from human biopsies from control (healthy) or DMD-diseased individuals were fixed with 4% paraformaldehyde/PBS and permeabilized in 1% tween 20/water. Antigen retrieval with sodium citrate buffer was conducted in a steamer for 30 min, and the slides were allowed to cool for 20 min. MuQ-FISH staining proceeded in the same fashion as for MuSCs, with the exception of washes. After the typical 6 washes with SSC buffers, two additional washes of 5 min each were completed with 0.25X SSC/0.1% tween 20 at 55ºC. After rinsing with PBS, slides were blocked with protein block (Dako) for 1 hr at room temperature. Antibodies against human CD45 (Dako, clone LCA; 1/250) or alpha smooth muscle actin (Abcam; clone E184, 1/200) were diluted in antibody dilutent (Dako) and incubated in a dark, humidified chamber overnight at 4ºC. Slides were washed the following day with PBS and incubated with secondary antibodies (Alexa Fluor 647-conjugated goat anti-rabbit IgG or Alexa Fluor 488-conjugated goat anti-mouse IgG; both at 1/250) in antibody dilutent for 1 hour at room temperature in the dark. After washing with PBS, coverslips were mounted with fluoromount G. MuQ-FISH imaging and analysis was completed on a single section, using methodology described previously in this manuscript.

C2C12 Cell Synchronization, Cell Cycle Analysis, and MuQ-FISH

The mouse myoblast cell line C2C12 was grown in DMEM supplemented with 10% FBS, 1X glutamax, and 1X anti-anti. For synchronization experiments, C2C12 cells were sparsely plated in 60mm dishes (for cell cycle) or 4 well chamber slides (for MuQ-FISH). The following day, cells were washed twice with PBS, and grown in either normal growth media (asynchronous), in DMEM containing only 1% serum for 36 hr (enriched in G1), or grown in growth media supplemented with 200 ng/mL nocodazole for 12 hr (enriched in G2/M), as described (Tintignac et al., 2004). Following incubations, cells were prepared for cell cycle analysis as described (Myer et al., 2011). Briefly, cells were trypsinized, washed with PBS, and fixed with cold 70% ethanol for 15 min. Cells were pelleted and resuspended in PBS, and treated with 40 µg/mL of boiled RNAse A. Cells were incubated at 37ºC for 20 min, and 10 µg/mL of Propidium iodide was added. Cells were analyzed on an LSR II flow cytometer. Figures were prepared with FLOWJO v.10.1. n=3 biological replicates of 20,000 events/sample. For MuQ-FISH, cells were imaged with a Nikon Plan Apo 40X/0.95 objective. The sum intensity values were used to generate figures.

Supplemental References

Mourkioti, F., Kustan, J., Kraft, P., Day, J.W., Zhao, M.M., Kost-Alimova, M., Protopopov, A., DePinho, R.A., Bernstein, D., Meeker, A.K.*, et al.* (2013). Role of telomere dysfunction in cardiac failure in Duchenne muscular dystrophy. Nat Cell Biol *15*, 895-904.

Myer, D.L., Robbins, S.B., Yin, M., Boivin, G.P., Liu, Y., Greis, K.D., Bhassi, el M., and Stambrook, P.J. Absence of polo-like kinase 3 in mice stabilizes Cdc25A after DNA damage but is not sufficient to produce tumors. Mutat Res *714*, 1-10.

Sacco, A., Mourkioti, F., Tran, R., Choi, J., Llewellyn, M., Kraft, P., Shkreli, M., Delp, S., Pomerantz, J.H., Artandi, S.E.*, et al.* (2010). Short telomeres and stem cell exhaustion model Duchenne muscular dystrophy in mdx/mTR mice. Cell *143*, 1059-1071.

Tintignac, L.A.J., Sirri, V., Leibovitch, M.P., Lécluse, Y., Castedo, M., Metivier, D., Kroemer, G., and Leibovitch, S.A. (2004). Mutant MyoD lacking Cdc2 phosphorylation sites delays M-phase entry. Mol Cell Biol *24*, 1809-1821.
