# Supplementary material for: Single Stem Cell Imaging and Analysis Reveals Telomere Length Differences in Diseased Human and Mouse Skeletal Muscles
Source: Stem Cell Reports. 2017 Sep 7;9(4):1328–41. doi: 10.1016/j.stemcr.2017.08.003 (PMC5639167; doi:10.1016/j.stemcr.2017.08.003)
Supplement: Document S2. Article plus Supplemental Information [file mmc2.pdf]

Stem Cell Reports
Resource

OPEN ACCESS

Single Stem Cell Imaging and Analysis Reveals Telomere Length Differences in Diseased Human and Mouse Skeletal Muscles

Elisia D. Tichy,[1] David K. Sidibe,[1] Matthew T. Tierney,[2] Michael J. Stec,[2] Maryam Sharifi-Sanjani,[1] Harish Hosalkar,[3] Scott Mubarak,[4] F. Brad Johnson,[5] Alessandra Sacco,[2] and Foteini Mourkioti[1,6,*]

[1]Department of Orthopaedic Surgery, Perelman School of Medicine, The University of Pennsylvania, 112A Stemmler Hall, 3450 Hamilton Walk, Philadelphia, PA 19104-6081, USA

[2]Development, Aging and Regeneration Program, Sanford Burnham Prebys Medical Discovery Institute, La Jolla, CA 92037, USA

[3]Joint Preservation Center, Tricity Medical Center, Joint Preservation & Deformity Correction Center & Traumatic Brain Injury Program, Paradise Valley Hospital, National City, CA 91950, USA

[4]Department of Orthopedic Surgery, Rady Children's Hospital, 3030 Children's Way, San Diego, CA 92123, USA

[5]Department of Pathology and Laboratory Medicine, Institute on Aging, Perelman School of Medicine, University of Pennsylvania, Philadelphia, PA 19104, USA

[6]Department of Cell and Developmental Biology, Perelman School of Medicine, The University of Pennsylvania, Philadelphia, PA 19104, USA

*Correspondence: fmour@mail.med.upenn.edu

http://dx.doi.org/10.1016/j.stemcr.2017.08.003

SUMMARY

Muscle stem cells (MuSCs) contribute to muscle regeneration following injury. In many muscle disorders, the repeated cycles of damage and repair lead to stem cell dysfunction. While telomere attrition may contribute to aberrant stem cell functions, methods to accurately measure telomere length in stem cells from skeletal muscles have not been demonstrated. Here, we have optimized and validated such a method, named MuQ-FISH, for analyzing telomere length in MuSCs from either mice or humans. Our analysis showed no differences in telomere length between young and aged MuSCs from uninjured wild-type mice, but MuSCs isolated from young dystrophic mice exhibited significantly shortened telomeres. In corroboration, we demonstrated that telomere attrition is present in human dystrophic MuSCs, which underscores its importance in diseased regenerative failure. The robust technique described herein provides analysis at a single-cell resolution and may be utilized for other cell types, especially rare populations of cells.

INTRODUCTION

Telomeres are long, repetitive DNA sequences (5′-TTAGGG-3′) that are present at chromosome ends (Collins, 2000). During each cycle of DNA replication, telomeres shorten, as DNA polymerases have no primers available to complex with and extend DNA (Ohki et al., 2001). Telomere shortening can also result from aberrant nuclease activity (Wu et al., 2012). Significantly eroded telomeres activate the DNA damage response, inducing cellular senescence and/or the activation of cell death processes (Shay and Wright, 2005). Cells have evolved mechanisms to combat such a dilemma. Classically, the action of telomerase (TERT), an RNA primer (TERC/TR), and accessory factors can extend telomere length in cells where these components are expressed and active (Sarek et al., 2015). The proper functioning of this pathway could play a crucial role in the regulation of stem cell aging and the prevention of the stem cell dysfunctional phenotype observed in degenerative disorders (Blasco, 2007b; Flores and Blasco, 2010).

Telomerase activity is most active during early development, after which the activity becomes reduced (Harley and Villeponteau, 1995). In the setting of degenerative disease, stem cells may lack the ability to extend telomere length, thus making them susceptible to premature dysfunction. Indeed, telomere shortening in relation to loss of self-renewal capacity has been reported in hematopoietic stem cells, induced pluripotent stem cells, and embryonic stem cells (Batista et al., 2011; Morrison et al., 1996; Niida et al., 2000). While telomere defects have been extensively studied in other systems and stem cell compartments (Flores et al., 2008), studies investigating telomere length dynamics in muscle stem cells (MuSCs) are lacking.

MuSCs, also known as satellite cells, are adult stem cells that localize between the sarcolemma and the basal lamina (Campbell and Stull, 2003). In undamaged muscle of adults, MuSCs remain quiescent (Brack and Rando, 2012). However, upon muscle injury a major tissue remodeling process occurs, leading to the activation and proliferation of resident MuSCs (Shi and Garry, 2006). Environmental cues lead to transcriptional activation of pathways inducing proliferation, differentiation, and fusion of differentiated progeny, which will comprise repaired muscle fibers (Wang and Rudnicki, 2011). Many muscle diseases, including muscle dystrophies such as Duchenne muscular dystrophy (DMD), present with multiple rounds of muscle damage and repair (Mann et al., 2011). Over time muscle weakness develops, resulting from a lack of complete regeneration (Wallace and McNally, 2009). A recent hypothesis to explain such an outcome is that the MuSC pool responsible for muscle regeneration gradually becomes less efficient at responding to and repairing damage as a result of stem cell defects (Dumont et al., 2015; Sacco et al., 2010).

This is an open access article under the CC BY license (http://creativecommons.org/licenses/by/4.0/).

However, it has not been studied whether critical telomere shortening in diseased MuSCs contributes to the progressive dysfunction that compromises their regenerative potential, in part due to the inability to quantitatively estimate telomere length in these cells. An optimized technique that is able to measure telomere length in a muscle cell type-specific way would be an invaluable tool to study the involvement of stem cells in the onset and progression of DMD as well as other skeletal muscle diseases.

Many methods exist to measure telomere length, either directly or indirectly (Montpetit et al., 2014). Direct methods such as telomere restriction fragment analysis (TRF) (Kimura et al., 2010) have several inherent shortcomings, including the requirement of a large sample size. Such assays are hindered by the low abundance of MuSCs within skeletal muscles (Morgan and Partridge, 2003). At the same time, human tissue is limited, and methods for propagating undifferentiated MuSCs in sufficient numbers to conduct TRF do not yet exist. While qPCR-based methods (O'Callaghan and Fenech, 2011) do not require as much starting material to perform, such assays can only measure the mean telomere length within the entire population of cells. Over the last few years it became widely accepted that MuSCs are a heterogeneous population (Ono et al., 2010; Tierney and Sacco, 2016). Therefore, when traditional qPCR-based methods are used to measure telomere length, information regarding individual cells within the population cannot be easily attained. Analyzing the expression or activity of TERT/TERC (Skvortsov et al., 2011) or using TRAP (telomerase repeated amplification protocol) activity assays (Fajkus, 2006) does not provide telomere information in a single-cell resolution context but rather in whole tissues. A robust technique for telomere length analysis is fluorescence *in situ* hybridization (FISH), in which a fluorescently labeled probe that is complementary to the telomere sequence is hybridized and fluorescence intensity is measured by immunofluorescence microscopy (Hande et al., 1999; Henderson et al., 1996). Historically, cells are treated with microtubule inhibitors to promote metaphase arrest, after which metaphase spreads are stained and analyzed (Hande et al., 1999). The caveats to such an approach are that only cultures with a high mitotic index could be analyzed (Ourliac-Garnier and Londono-Vallejo, 2011), and a large number of cells are needed (Howe et al., 2014), making this method challenging for quiescent stem cells directly isolated from a tissue. Assessment of telomere length in interphase cells using *in situ* hybridization and digital fluorescence microscopy on tissue sections (de Pauw et al., 1998; O'Sullivan et al., 2004) is challenging due to the fact that MuSCs are sparse within skeletal muscles. In fact, our own attempts to measure telomere length by adapting FISH analysis on skeletal muscle sections coupled with the MuSC-specific marker, Pax7, was abandoned due to the sparse number of MuSCs per section. Flow-FISH is a version of FISH that can be combined with the detection of cell surface markers and examines mean values of telomere length in populations of non-dividing cells in interphase (Hultdin et al., 1998). However, most available flow-FISH protocols focus on analysis of hematopoietic cells and cultured cells (Baerlocher et al., 2006), and its use with other tissues and cell types has not been reported. A second drawback of flow-FISH is that it only provides mean telomere length values per cell, in contrast to the quantification of individual telomere foci signals in the case of microscopy-based methods (Meeker et al., 2002). Importantly, in our experience, the heat denaturation step, an absolute requirement of this method (Lauzon et al., 2000), compromises the cell surface antigens used for MuSC isolation.

Although all previous methods have been utilized for a variety of tissues and cells, we desired a better quantitative microscopy-based method that will be applicable for use with the rare population of stem cells present in skeletal muscle tissues. Here, we describe a protocol, designated henceforth MuQ-FISH (muscle quantitative fluorescence *in situ* hybridization), which first enriches MuSCs prior to the application of a modified FISH method that is optimized for MuSCs of mouse (mMuSCs) and human (hMuSCs) origin. The development of MuQ-FISH allows for quantification of both telomere length and number in prospectively isolated MuSCs, regardless of species origin or disease state. MuQ-FISH has many differences compared with other FISH methods, including MuSC isolation and processing, telomere staining, and analysis. After validating the MuQ-FISH method using mice lacking the RNA component of telomerase (TERC/TR), we showed that there were no telomere length differences in MuSCs isolated from uninjured young and old mice. Additionally, telomere shortening became evident in MuSCs isolated from dystrophic mice. Interestingly, telomere shortening was also discovered in hMuSCs from DMD patients compared with healthy aged-matched human cohorts. Thus, the method described herein could become a standard tool to measure telomere length in other stem cell diseases and disorders, especially muscle-based diseases, and can potentially be expanded for use in other resident muscle cell types.

RESULTS

Validation of MuQ-FISH

The effect of telomere length on maintaining tissue homeostasis and stem cell function has been studied in many tissues (Flores and Blasco, 2010; Flores et al., 2008; Morrison et al., 1996; Sekulovic et al., 2011; Shekhani

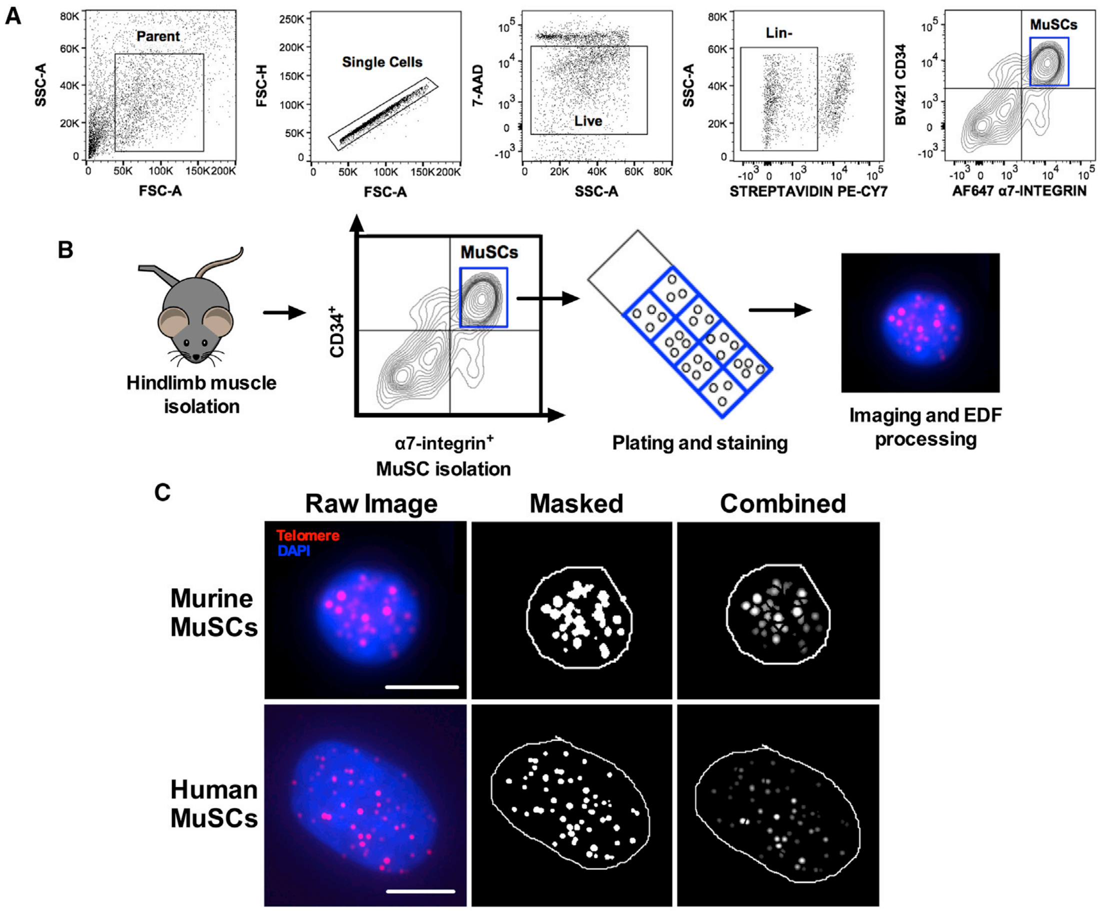

Figure 1. Isolation of Mouse Muscle Stem Cells and Processing for Telomere MuQ-FISH Analysis

(A) Flow-cytometry scatterplots of the gating strategy used for isolation of MuSCs from C57BL/6 mice. Live cells were 7-AAD^{-} and MuSCs were defined as CD11b^{-}CD31^{-}Sca1^{-}CD45^{-}, CD34^{+}, α7-integrin^{+}. See also Tables S1 and S2 for information regarding antibodies and filter sets used. See also Figure S1.

(B) Schematic of telomere staining protocol of mMuSCs. EDF denotes extended depth of focus, which is a stacking technique used to create an in-focus flattened image. See also Figure S2.

(C) Left: raw EDF representative image of stained MuSCs from uninjured wild-type mice (top) or human patients (bottom). Red, telomere probe; blue, DAPI-stained nuclei. Scale bars represent 5 μm for murine MuSCs and 10 μm for human MuSCs. Middle: software analysis image of bounded nuclei depicting telomere foci. Right: processed cells in which telomere intensity is measured after manual separation of conjoined particles.

et al., 2016). However, in the skeletal muscle field, existing methods are not well suited for stem cell analysis due to technical and biological limitations of the muscles (rare population, fragile cells, no MuSC expansion, and small tissue sample availability). Notably, several of the steps used in other FISH protocols that work well for other tissue stem cells (Flores et al., 2008) are not favorable for MuSC staining. To measure telomere length in a MuSC-specific way and avoid previous limitations, we developed MuQ-FISH. More specifically, we first isolated MuSCs from uninjured wild-type mice by fluorescence-activated cell sorting (FACS) (Figure 1A) based on cell surface markers, as described by Sacco et al. (2008). FACS was followed by a short plating (8–10 hr) and fixation with 4% paraformaldehyde (Figure S1A), prior to staining for MuQ-FISH (Figure 1B). In our experience, the plating of cells prior to MuQ-FISH does not alter key criteria, including the differentiation state or viability of MuSCs (Figures S1B–S1F). Fixed cells were then stained using a Cy3-conjugated telomere sequence-specific probe labeled at the C terminus, followed by nuclear staining with DAPI. It was critically important that during image acquisition for each experiment, the exposure settings were kept the same for each color channel and that the signal intensities were not too low or too high (Figure S2). For mMuSCs, z stacks were taken for each color channel and combined and flattened using an

extended depth of focus/field (EDF) algorithm (Figure 1B). EDF is a digital method of processing a series of images (optical sections) focused at a range of depths. This EDF method results in a single image, with all areas in focus (Hovden et al., 2011). We found that the use of EDF processing in MuQ-FISH considerably improves the image quality and increases the accuracy of telomere foci measurements. Images were scaled to 16 bit and monochrome images for each channel were opened in the telomere analysis software, Telometer, and processed to subtract background noise. DAPI-stained nuclei were manually encompassed, and this region was used to determine a region of interest, in which telomere signal intensities were measured (Figure 1C). Telometer then compiles data from intensity measurements, which can be used for different analyses. Notably, the software is capable of discerning telomere length of cells in different stages of the cell cycle and normalizing the data, based on DNA content (Figure S3). The protocol for measuring telomere intensities is identical for hMuSC analysis (Figure 1C), with the exception that z stacks are not required for analysis due to the more planar morphology of hMuSCs in culture.

Data obtained from the telomere software of MuQ-FISH can be analyzed in different ways. Common approaches include measuring the total signal intensities of the telomere reads and dividing that value by the total signal intensity of the DAPI (referred to as sum intensity) or identifying the mean intensity of the telomere signal divided by the total signal intensity of the DAPI (referred to as mean intensity). To delineate rare populations of cell data that may be buried by the average telomere analysis described, MuQ-FISH data can also be broken down and plotted as histograms, using the same datasets for sum intensity or mean intensity. Finally, information regarding the number of telomeres, as defined by the individual signal reads, can be obtained, which can also be depicted as an average or as a histogram revealing the telomere length distribution within each cell population of a given genotype or condition.

Using our newly optimized methodology, we validated our approach by FACS-isolating MuSCs from wild-type mice, and from mTR^{G1} and mTR^{G3} mice, the latter of which should globally present with shorter telomere length (Blasco et al., 1997). After collecting similar numbers of MuSCs from all groups and processing cells for MuQ-FISH (Figures 2A and 2B), we interrogated the telomere length and number of telomere foci in all groups. Applying the analysis methods described above, we found significant shortening of telomere length when either the sum or the mean intensity of the telomere signal was measured between the different genotypes (Figures 2C and 2D). To examine differences in the number of foci with detectable telomere repeat tracks in mMuSCs, we queried the number of telomere foci per nucleus and found significantly fewer telomere signals only in the mTR^{G3}-derived MuSCs (Figure 2E), in accordance with lack of detectable telomere repeats in late $mTR^{-/-}$ generations (Blasco et al., 1997). This analysis provides a biological validation of the MuQ-FISH method as a protocol that measures telomere length and number of telomere foci in stem cells derived from skeletal muscles.

MuQ-FISH Analysis of Young and Old MuSCs

Telomere shortening is reported to be a hallmark of aging in some organisms (Blackburn et al., 2015; Harley et al., 1990; Wong et al., 2003). To identify the effects of aging on the telomere length and number in MuSCs, young (~2 months old) and old (~25 months old) wild-type MuSCs were FACS-sorted from mice in the absence of any induced injury and processed for MuQ-FISH (Figures 3A and 3B). This analysis revealed no major differences in either the telomere length or the number of telomere foci between MuSCs derived from young and old mice (Figures 3C and 3D). To further assess the data, we presented telomere length values as histograms to delineate any rare cell populations that were lost when total data were analyzed. Using this approach, no significant shortening of telomeres was observed during aging (Figures 3C and 3D), suggesting that in the absence of muscle injury, telomere length is stable in aging murine MuSCs.

MuSCs Isolated from a Dystrophic Mouse Model with a Severe Phenotype Have Shortened Telomeres

In hematopoietic and lung disease states, telomere attrition in stem cells affects their regenerative capacity (Alder et al., 2015; Armanios, 2013). To query telomere length of MuSCs derived from a skeletal muscle disease condition, we examined telomere differences in young (3–4 months old) control and aged-matched dystrophic mice. Wild-type, mdx (carrying a dystrophin mutation), mTR^{G2}, and mdx/mTR^{G2} (combined dystrophin and TERC mutation) mice were first queried for differences in cell number during FACS isolation. mdx/mTR^{G2} mice exhibit a more severe muscle phenotype than mdx/mTR^{G1} mice or mdx alone (Mourkioti et al., 2013), while mTR^{G2} mice do not exhibit muscle defects under non-injury conditions (Sacco et al., 2010). While similar numbers of MuSCs were isolated between wild-type, mdx, and mTR^{G2} mice (Figure 4A), a comparison of mTR^{G2} mice with mdx/mTR^{G2} mice did show a decrease in MuSC numbers in the dystrophic mice, consistent with the reported difference in stem cell reserves available for regeneration during the progression of the dystrophic disease (Sacco et al., 2010).

We then processed isolated mMuSCs from wild-type, mdx, mTR^{G2}, and mdx/mTR^{G2} mice for MuQ-FISH (Figure 4B). We showed variable shortening of telomeres

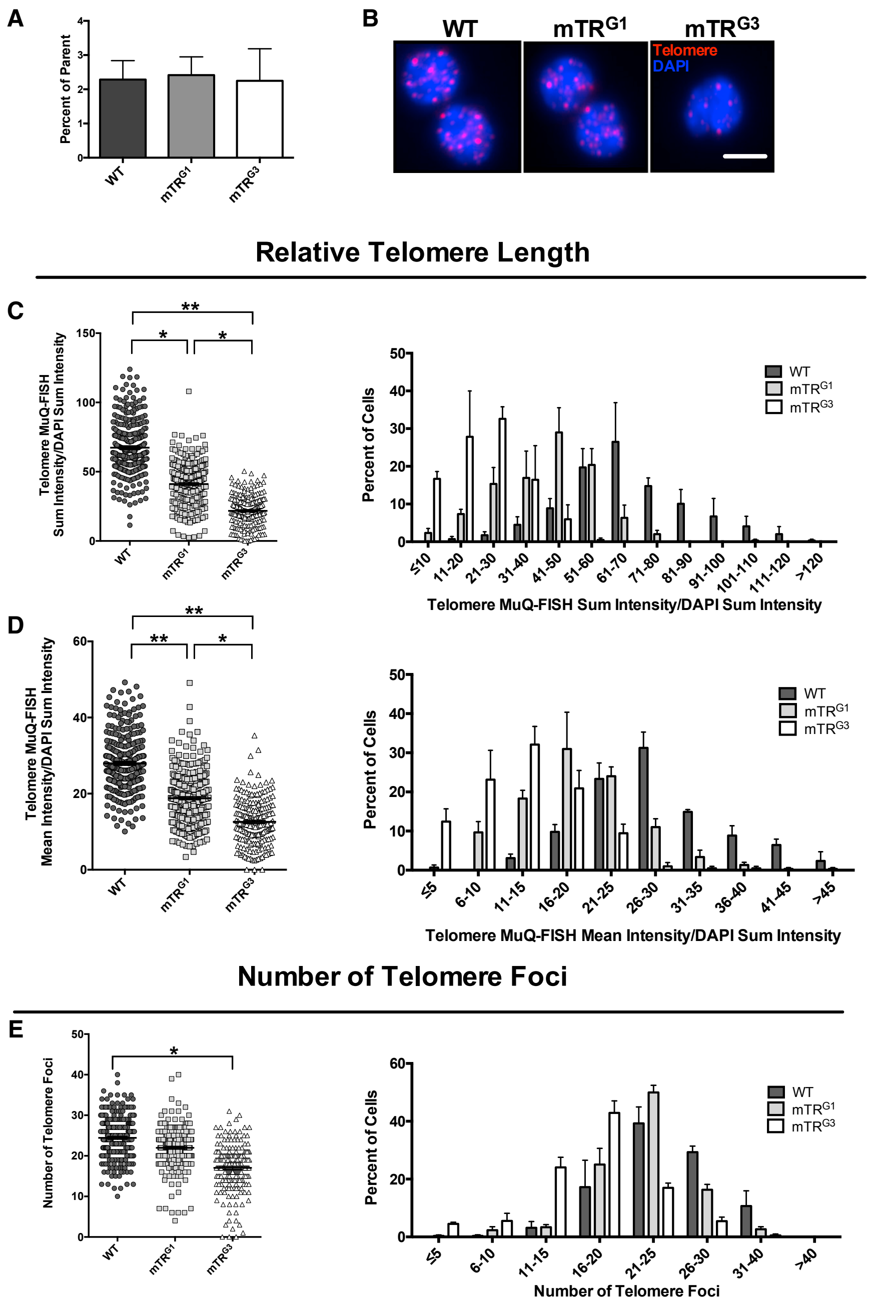

Figure 2. Comparative Analysis of Telomere Length and Number in Different Generations of TERC/TR Knockout Mouse-Derived MuSCs

(A) Number of MuSCs isolated from 20,000 total events. Data displayed are the percentage of $CD34^+/\alpha7$-integrin$^+$ MuSCs that comprised the total number of cells in the parent gate. Data are displayed as the mean ± SEM from n = 2–3 female mice per group of similar age.

(B) Representative images of cells processed for MuQ-FISH. Scale bar, 5 μm.

(C) Left: sum intensity of telomere signals divided by DAPI sum intensity. $n \geq 90$ cells were analyzed per mouse, with n = 2–3 mice per category. Bars represent mean ± SEM. $^*p \leq 0.05$, $^{**}p \leq 0.01$. Right: histogram depiction of sum intensity measurements (mean ± SEM).

(D) Left: data representation of telomere mean intensity divided by DAPI sum intensity. Right: histogram representation of data in left panel. $^*p \leq 0.05$, $^{**}p \leq 0.01$.

(E) Left: relative number of telomere foci in wild-type, mTR^{G1}, and mTR^{G3} mice. Right: histogram representation of number of telomere foci. $^*p \leq 0.05$.

WT, wild-type.

between these MuSCs by plotting sum intensity values, with longer telomeres found in the wild-type MuSCs, with the mdx/mTR^{G2} MuSCs displaying shorter telomeres (wild-type > mdx > $mTR^{G\ 2}$> mdx/mTR^{G2}; Figure 4C). Furthermore, the distribution of telomere length (histogram) shows a more profound shift to shorter telomere length of the mdx/mTR^{G2} MuSC population (red in Figures 4C and S4A), suggesting that the telomere shortening observed in mdx/mTR^{G2} MuSCs is not solely the result of the mTR^{G2} genetic manipulation, but rather seems to be an additive effect of the combination of mdx and mTR^{G2} in these cells. Similar reductions in telomere length were found using confocal microscopy (Figure S4B). Further characterization of mMuSC telomere dynamics was carried out, whereby numbers of foci were examined between diseased and control mMuSCs. Interestingly, the number of telomere foci was very dynamic between all groups examined (Figure 4D). Overall, these data demonstrate that MuSCs from a murine model with severe muscular dystrophy exhibit extensive telomere shortening.

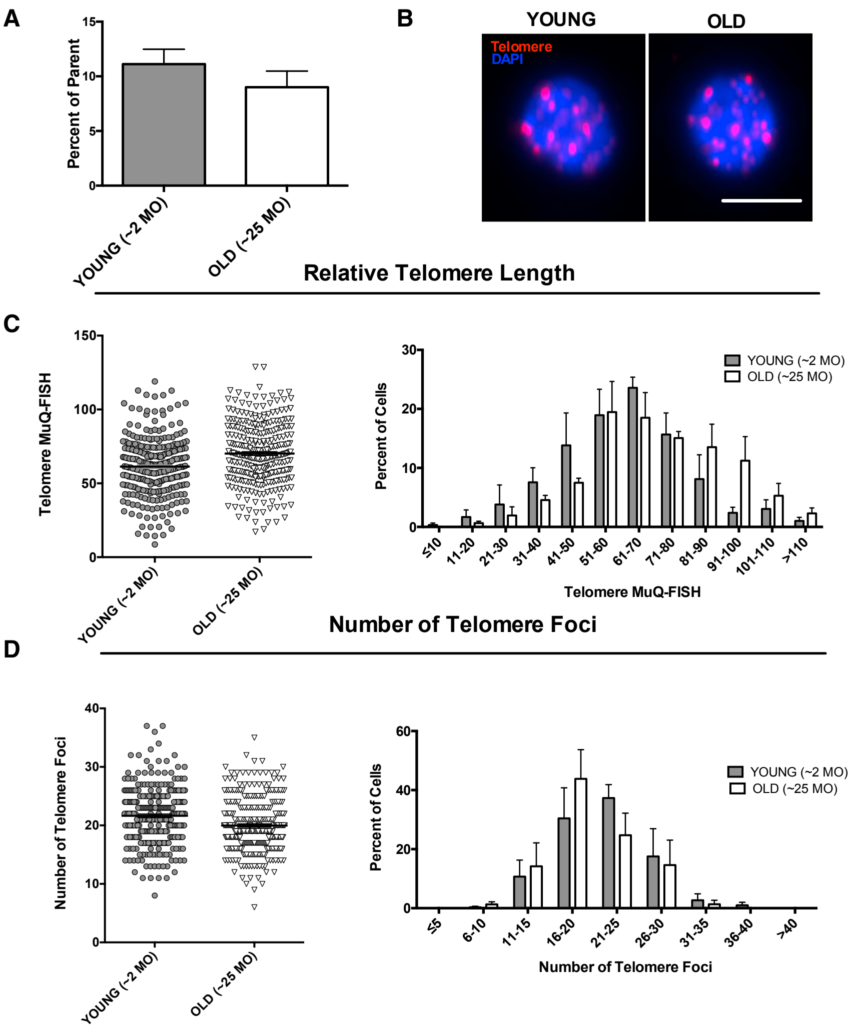

Figure 3. Comparative Analysis of Telomere Length and Number in Young and Old Murine MuSCs by MuQ-FISH

(A) Number of MuSCs isolated from 50,000 total events. Data are displayed as the mean ± SEM from n = 3 male mice per group of identical age.

(B) Representative images of cells processed for MuQ-FISH. Scale bar, 5 μm.

(C) Left: sum intensity of telomere signals divided by DAPI sum intensity. n ≥ 90 cells were analyzed per mouse, with n = 3 mice per category. Bars represent mean ± SEM. Right: histogram depiction of sum intensity measurements.

(D) Left: relative number of telomere foci in wild-type young and old mice. Right: histogram representation of number of telomere foci.

Dystrophic hMuSCs Exhibit Shortened Telomeres

To determine whether the telomere length observations in diseased mMuSCs recapitulate what happens in the human dystrophic condition, we collected hMuSCs from the biopsies of three healthy and three DMD-diseased teenage individuals (Figure 5A), and FACS isolation revealed that adequate hMuSC numbers were procured (Figure 5B). Following a short culture that allowed attachment of cells on slides (Figures 5C and S5), hMuSCs were processed for MuQ-FISH (Figure 6A) and telomere lengths and number were calculated. This analysis revealed that telomere length was significantly shorter in DMD-diseased hMuSCs compared with healthy MuSCs, with a clear shift toward shorter telomeres in the distribution representation (Figure 6B) without a significant loss in telomere foci (Figure 6C). These data support the notion that accelerated telomere shortening in the setting of muscular dystrophy is also a characteristic of hMuSCs, extending this finding to the human disease.

To address the specificity of hMuSC-telomere shortening, we conducted MuQ-FISH on patient muscle biopsies, followed by staining for the common leukocyte antigen, CD45 (Thomas and Lefrancois, 1988) as well as for α-smooth muscle actin, which labels smooth muscle cells (Skalli et al., 1987). In both cases telomere lengths, as assayed by MuQ-FISH, are not significantly different between healthy and DMD patient samples that stained positively for these markers (Figures S6 and S7). This result argues against ubiquitous telomere shortening in other skeletal muscle-derived cells other than MuSCs and suggests that the telomere shortening, which we observed in DMD patient-derived MuSCs, is likely the result of the disease process.

DISCUSSION

Telomere shortening can occur in actively proliferating cells, cells with aberrant nuclease activity, or cells experiencing

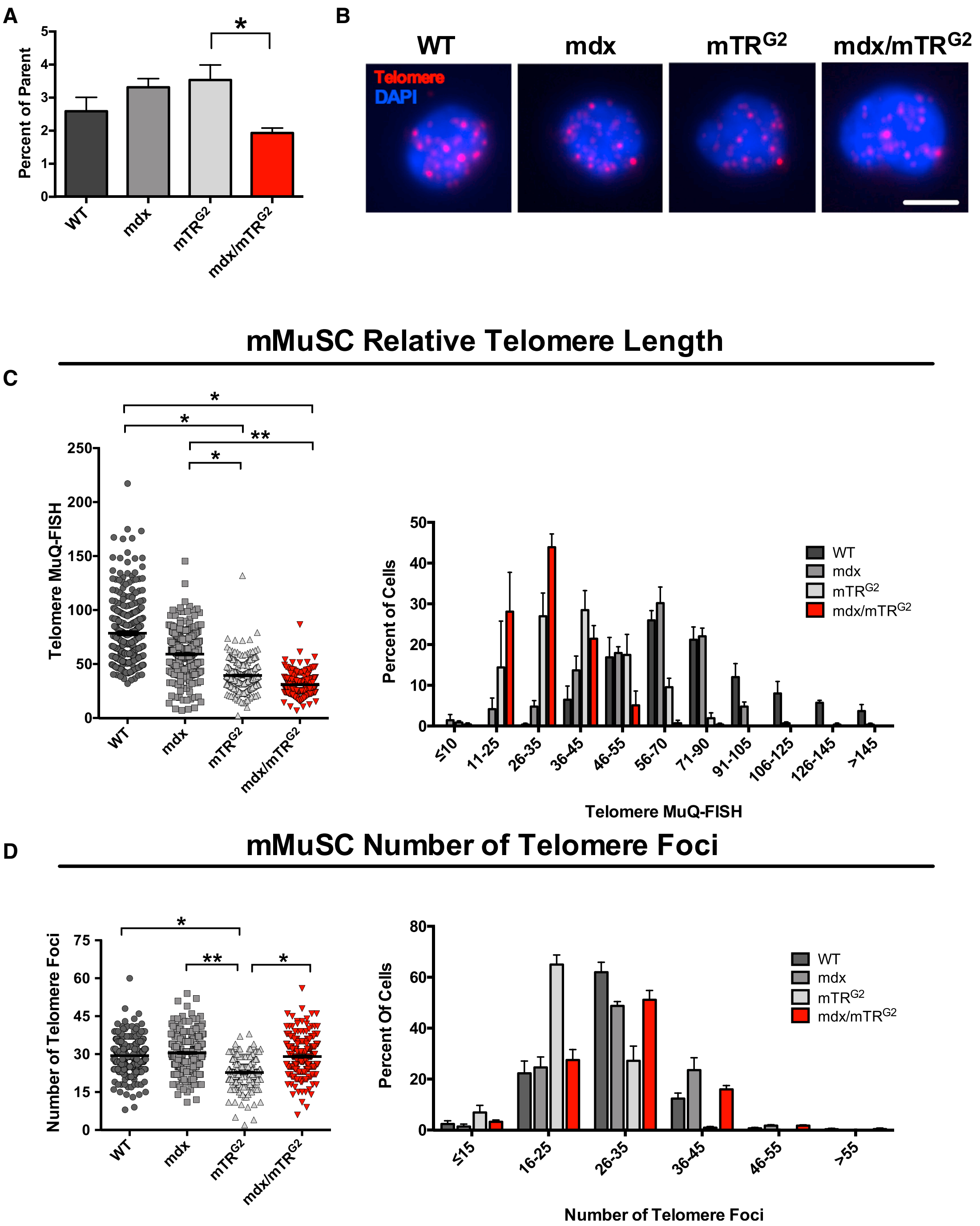

Figure 4. Muscle Stem Cells from Dystrophic Mice Exhibit Significant Telomere Shortening Compared with Control Mice

(A) FACS analysis of the number of mMuSCs isolated from mice of different genotypes, represented as percentage of the parent gate. Data are displayed as mean ± SEM. $*p \leq 0.05$.

(B) Representative images of MuQ-FISH from mMuSCs of different genotypes. Scale bar, 5 μm.

(C) Left: plotted data of telomere sum intensity calculation. Bars represent mean ± SEM. $*p \leq 0.05$, $**p \leq 0.01$. Right: data plotted as histograms. Note the shift toward the shortest telomeres in the mdx/mTR^{G2} MuSCs. n = 3 mice per genotype and n $\geq$ 70 cells per mouse. See also Figure S4.

(D) Left: dot plot showing number of MuSC telomere foci between genotypes. Bars represent mean ± SEM. $*p \leq 0.05$, $**p \leq 0.01$. Right: histogram depiction of data shown on the left.

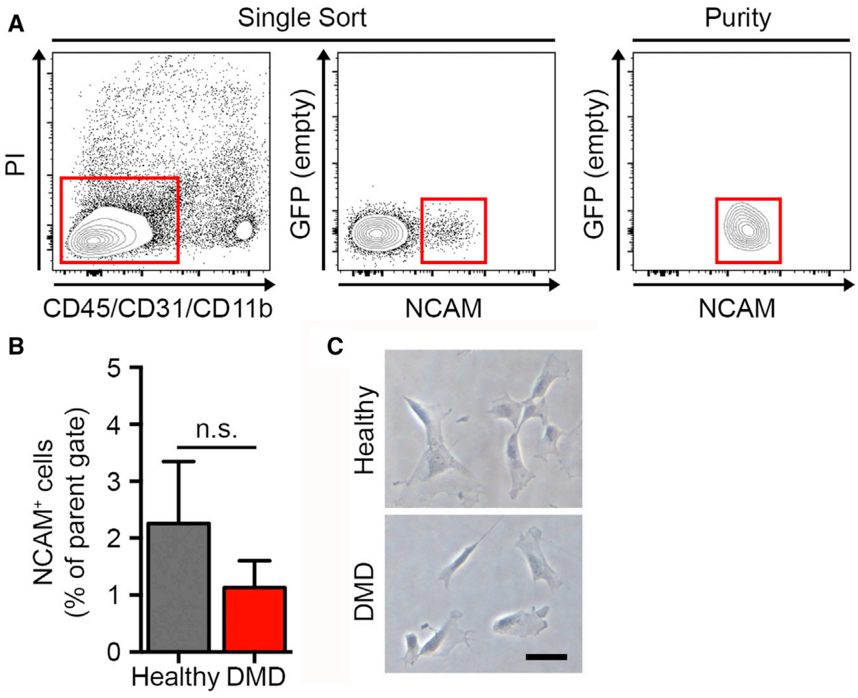

Figure 5. Isolation and Quantitation of Human Muscle Stem Cell Number

(A) FACS plots of the gating strategy used for the isolation of human healthy and dystrophic MuSCs. Mononucleated cell suspensions from human muscle biopsies were prepared and cells expressing cell surface markers $CD45^{-}CD11b^{-}CD31^{-}$ and $NCAM^{+}/CD56^{+}$, which we define as hMuSCs, were fractionated.

(B) Percentage of $NCAM^{+}/CD56^{+}$ cells (hMuSCs) that comprise the parent sort gate. Data are displayed as the mean ± SEM from n = 3 human biopsies per group. n.s., not significant.

(C) Representative images of cultured hMuSCs isolated from healthy (upper) and DMD (lower) individuals. Scale bar, 50 μm.

See also Figure S5.

DNA damage, particularly in those lacking functional telomerase (d'Adda di Fagagna et al., 2003; Herbert et al., 1999). Significant telomere attrition leads to the activation of DNA damage signaling pathways (Viscardi et al., 2007), which induces cell cycle arrest, senescence, and/or cell death. Other consequences of telomere shortening include the alteration of gene expression (Ye et al., 2014), induction of epigenetic modifications and promoter methylation (Blasco, 2007a) and altered gene silencing near the telomere (Guan et al., 2013), as well as the alteration of gene expression megabases away from telomeres (Robin et al., 2014). These effects, either individually or in combination, contribute to stem cell dysfunction.

Stem cell functions decrease in many tissue types during aging, which often correlates with telomere shortening (reviewed in Sharpless and DePinho, 2007). Interestingly, short telomere lengths limit stem cell numbers and function in hematopoietic cells (Armanios, 2013), while short telomeres can contribute to stem cell failure in the lung (Alder et al., 2015). Moreover, telomere shortening is suggested to act as a biological clock that counts cell divisions to regulate the number of self-renewing divisions within each differentiation state in hematopoietic cells (Morrison et al., 1996). In the skeletal muscle system, the progressive loss of the mMuSC reserve plays a major role in determining the severity of the dystrophic phenotype (Dumont et al., 2015; Sacco et al., 2010). However, due to the lack of a proper method to measure telomere length in MuSCs, all previous analyses were not performed in MuSCs but rather in their differentiated myoblast derivatives (Ramunas et al., 2015; Sacco et al., 2010) or in whole muscle extracts (Decary et al., 2000). The technique described here is specific for the skeletal muscles and has many differences among previously reported FISH methods in other tissues, including modifications of the probe employed, the cell isolation and fixation method, the optimized staining procedure, and the incorporation of telomere software for unbiased analysis. MuQ-FISH allows for telomere foci number and length assessment of individual cells within the population and is applicable to both muscle cells cultured shortly *in vitro* and/or freshly isolated from *in vivo* sources. More specifically, MuQ-FISH has several advantages compared with other methods: (1) the analysis of telomere length can occur at the single-cell level, such that data from scarce populations are not diluted and lost; (2) no radioactive compounds are required; (3) the assay is optimal for analyzing cells where only a small amount of material can be obtained, as is generally the case for cells derived from human biopsies; (4) this protocol uses common and/or inexpensive equipment to complete; and (5) analysis of both proliferating and non-proliferating or quiescent cells is possible. Thus, factors influencing proliferation and/or cell homeostasis and their effects on telomere attrition can all be tested including, but not limited to, genetic manipulation, aging, and culture conditions such as oxygen tension, substrate rigidity, and the alteration of growth factors.

The current study established a new approach in a translationally relevant biological system, by measuring

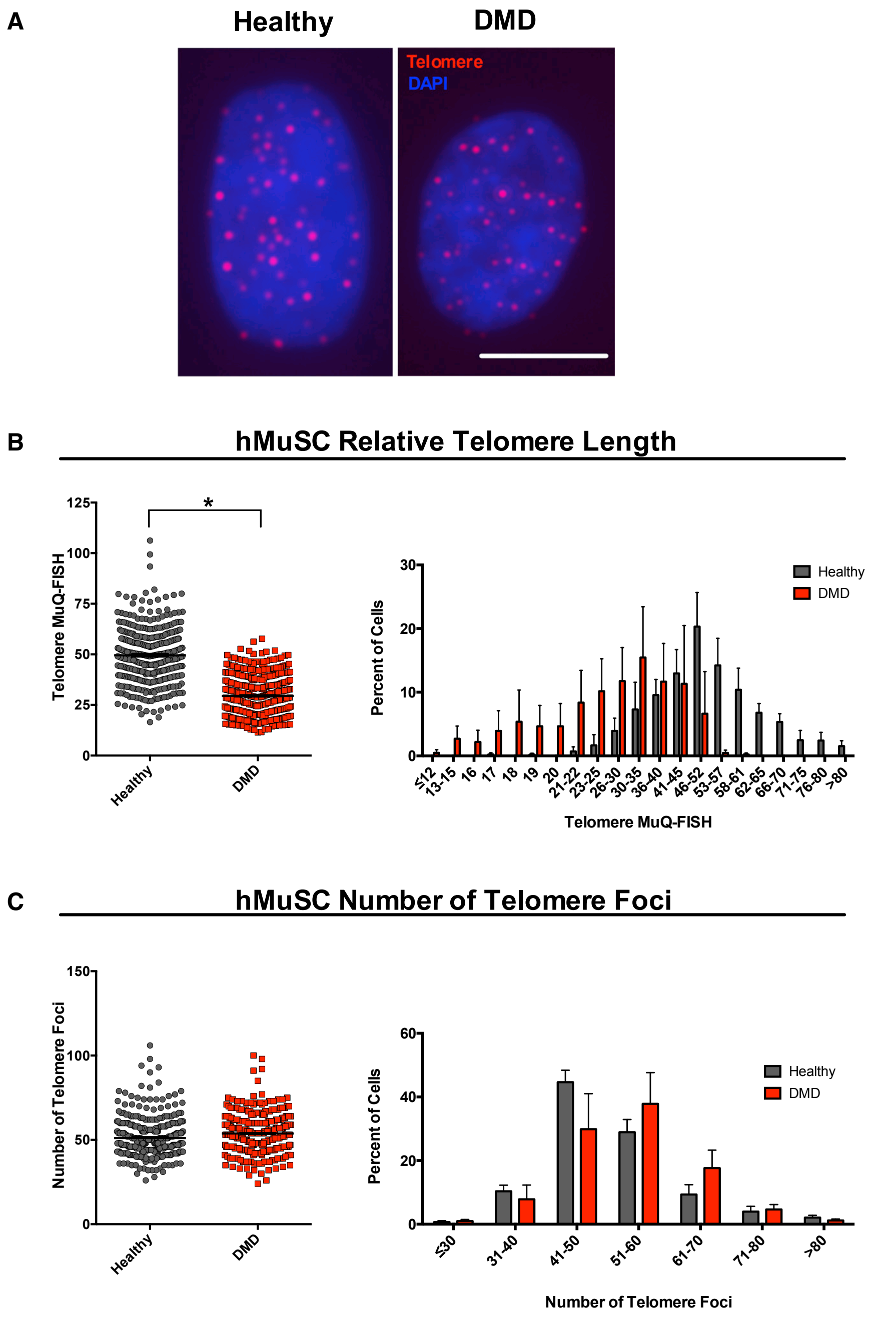

Figure 6. MuQ-FISH Analysis Reveals Significantly Shortened Telomeres in hMuSCs Isolated from DMD Patients Compared with Age-Matched Healthy Individuals

(A) Representative images of telomere MuQ-FISH in healthy and dystrophic hMuSCs. Scale bar, 10 μm.

(B) Quantification of the average telomere length per nucleus (left) and the sum intensity distribution presented as a histogram (right). Note the clear shift toward shorter telomere length in the diseased population. n = 3 human biopsies per group and n $\geq$ 100 cells per patient were analyzed. Bar represents mean ± SEM. *p $\leq$ 0.05. See also Figures S6 and S7.

(C) Left: measurements of relative number of telomere foci in healthy and dystrophic hMuSCs. Bars represent mean ± SEM. Right: distribution of number of telomere foci shown as histograms.

telomere length in MuSCs derived from young and old mice as well as in healthy and dystrophic mMuSCs and hMuSCs. The rationale for such a study is that in many stem cell systems, telomere shortening often occurs during aging (Armanios et al., 2009), as well as in many degenerative diseases (Calado and Young, 2009; Harley, 2005). Our analysis revealed that telomere length of mMuSCs deriving from old wild-type mice did not shorten when compared with young wild-type mMuSCs. Although telomere shortening is major determinant of aging in other systems (Blackburn et al., 2015; Harley et al., 1990; Wong et al., 2003), our finding of stable MuSC telomere length is in agreement with other reports measuring telomere length in whole muscle fibers from young and aged mice (O'Connor et al., 2009) as well as telomere length findings in skeletal muscles of non-human primates (Gardner et al., 2007).

Muscle wasting, caused by myofiber fragility, is exacerbated by intrinsic MuSC dysfunction, leading to impaired regeneration (Chang et al., 2016). Our initial MuQ-FISH studies were extended to further examine telomere

dynamics in age-matched MuSCs derived from dystrophic mice undergoing chronic regeneration, as well as in genotype control MuSCs. Although mTR^{G2} mice have no reported skeletal muscle phenotype, their MuSCs exhibited significantly shorter telomeres compared with wild-type controls. This result validates the MuQ-FISH method and further demonstrates the sensitivity of the assay in evaluating premature telomere length in a stem cell-specific way. Intriguingly, we show that telomere shortening is present in mdx/mTR^{G2} MuSCs and to a lesser extent in mdx MuSCs. The order of telomere shortening in these groups correlates with the severe penetrance of the dystrophic phenotype observed in mdx/mTR^{G2} compared with mdx mice (Sacco et al., 2010). These findings support the notion that telomere shortening contributes to the stem cell dysfunction and impaired regeneration observed in muscular dystrophy. Our results of dissimilar numbers of foci between dystrophic and wild-type MuSCs are important to consider in light of previous studies that show an increased percentage of chromosomes with telomere signal free ends in dystrophic myoblasts (Sacco et al., 2010). In contrast to the extensively cultured myoblasts utilized in the previous study, MuQ-FISH is able to detect telomere foci in MuSCs soon after their isolation from dystrophic muscles, which may reconcile the signal-intensity differences observed between the two designs.

Although much is known about MuSCs and the tissue-regenerative abilities in these murine models, very little information has been obtained regarding humans. Current findings of regeneration processes in human muscle diseases are limited, due to the fact that hMuSC analysis has lagged behind. Using MuQ-FISH, we have successfully isolated dystrophic and aged-matched healthy MuSCs from human muscle biopsies and demonstrated premature telomere shortening in MuSCs DMD patients, while the numbers of telomere foci were not different, consistent with our mouse findings. Extension of MuQ-FISH to other resident cell types in human muscle sections demonstrated that telomere attrition occurs in MuSCs but that it is not an inherent process in non-skeletal muscle cell types. Thus, the premature telomere shortening observed in DMD implies an intrinsically limited functional potential of human dystrophic MuSCs, and the expansion of dystrophic MuSCs as a therapeutic strategy may have a limited window of effectiveness before critical telomere shortening triggers replicative senescence and regenerative failure in DMD muscles.

In summary, we provide an optimized and validated assay to detect telomere length in stem cells isolated from skeletal muscles of mice and humans. Analyzing the role of telomere length in hMuSCs using the MuQ-FISH method described here will allow for a better understanding of the molecular genetic events that, in relation to telomere dysfunction, may lead to the delineation of muscle regeneration limitations in different human skeletal MuSC disease etiologies. Importantly, utilizing MuQ-FISH will allow for further investigation of the developmental and self-renewal processes and pathways involved in stem cell dysfunction in various skeletal muscle disorders. Diseases of particular interest include other muscular dystrophies, muscle atrophy, sarcopenia, myosarcomas, and chronic muscle injuries. Moreover, using this method we can extend the analysis to other cells isolated from the same tissues, with particular interest in cell types that influence MuSC function and muscle regeneration, such as fibroadipogenic progenitor cells (Joe et al., 2010). A better understanding of the molecular players and the exact mechanisms that regulate stem cell function, telomere length, and skeletal muscle homeostasis in humans should constitute an important advance in the field of regenerative medicine.

EXPERIMENTAL PROCEDURES

Mice

Mice were housed and bred in accordance with Institutional Animal Care and Use Committee guidelines outlined by the University of Pennsylvania. Additional information can be found in Supplemental Experimental Procedures.

Human Muscle Biopsy Procurement

Human muscle biopsies from healthy and DMD patients were obtained from the lower extremity muscles during surgical procedures as part of the patient's clinical care plan at Rady's Children's Hospital, San Diego. Written informed consent from the parent or guardian was obtained for all subjects. The protocol was approved by the University of California, San Diego Human Research Protectants Program and Institutional Review Board in accordance with the requirements of the Code of Federal Regulations on the Protection of Human Subjects. Three healthy and three DMD-affected biopsies were collected for this study.

Murine-Derived MuSC Quantitative Fluorescence *In Situ* Hybridization

After sorting, a minimum of 2,000 mMuSCs were plated onto laminin-coated (Sigma-Aldrich) 8-well chamber slides (Nunc; Labtek II, Thermo) in 50 μL of myoblast medium (Figure S1) and left at room temperature overnight, uncovered to dry onto the slides. This step results in the retention of more MuSCs on the slide following the staining procedure. The next day, cells were fixed in 4% paraformaldehyde/PBS for 15 min and rinsed once with PBS. Cells were permeabilized with 0.1% Tween 20/PBS for 5 min and washed with PBS twice for 3 min each. Cells were treated with 100 μg/mL pre-boiled RNAse A for 20 min at 37°C, followed by three PBS washes of 5 min each. Slides were dried. The Cy3-conjugated PNA telomere probe (1/300 dilution of 50 μM stock of TelC probe; F1002; PNAbio; 5′-CCC TAA CCC TAA CCC TAA-3′) was prepared in MuQ-FISH buffer (60% formamide, 5%

of 10× blocking agent for nucleic acid hybridization [not diluted to 1×; Roche], 2% 1 M Tris [pH 7.5], all in water). The probe and slide were preheated to 86°C for 10 min before the probe was added to the slides. After probe addition, slides were covered and incubated for an additional 10 min at 86°C, prior to cooling overnight at room temperature in the dark. The following day, slides were washed twice in a stepwise fashion with pre-warmed (55°C) 2× saline sodium citrate (SSC)/0.1% Tween 20, 1× SSC/0.1% Tween 20, and 0.5× SSC/0.1% Tween 20. Chamber slides were rinsed with PBS, disassembled, and coverslips were mounted with Fluromount G with DAPI (SouthernBiotech).

Human-Derived MuSC Quantitative Fluorescence *In Situ* Hybridization

hMuSCs were cultured in DMEM-F12 with 15% fetal bovine serum and 1× antibiotic-antimycotic. For human MuQ-FISH staining, hMuSCs were plated and grown overnight in a 37°C/5% CO_2 humidified incubator on collagen-coated 8-well chamber slides (Nunc; Labtek II, Thermo) and processed as stated in the mMuSC protocol.

Image Acquisition

Images of mMuSCs were taken using a Nikon eclipse 90*i* wide-field epifluorescence microscope equipped with a Prior Proscan III motorized stage, a Photometrics Coolsnap HQ2 14-bit digital camera, and a Nikon 100×/1.40 Plan Apo VC objective. Five to eight z stacks, which corresponded to the lower and upper boundaries of DAPI signal per nucleus, were taken for both the DAPI and Cy3 channels, at a 1-μm step. Samples are then imaged to determine each channel's exposure time in a way that situates the intensities for all samples in the mid-intensity range (Figure S3). This control prevents overexposure or loss of signal detection with the selected exposure times during sample imaging (Figure S3A). Once an optimal exposure time is defined (Figure S3B), image acquisition utilizes the same exposure settings for the Cy3 (telomere) and DAPI signals for imaging of individual experiments. For each experiment, the intensity settings are not changed between sample groups. Images are taken without binning. Z stacks are combined using the extended depth of focus/field (EDF) option in the Nikon elements software. Images of hMuSCs were taken with a Nikon eclipse Ni-U wide-field epifluorescence microscope equipped with a Nikon Qi1Mc 14-bit camera and a Nikon 100×/1.24 Plan Apo objective. A minimum of 30 cells per sample set is required for analysis; however, the more cells imaged, the more accurate the resultant data are representative of the source. Regardless, imaging must take place in the same time window for each experiment, and combination of multiple experiments performed on different days is not recommended, due to the inherent caveat of fluorescent intensity measurements differing between experiments.

Telomere Analysis

Telomeres were analyzed with the investigators blinded to genotypes and/or conditions using open-source software (Telometer; http://demarzolab.pathology.jhmi.edu/telometer/), as previously described (Meeker et al., 2002; Mourkioti et al., 2013). Other telomere-measuring software could also be used for such analysis, including but not limited to Telomap (Flores et al., 2008), TFL-Telo (Poon and Lansdorp, 2001), Tissue-Telo (Aida, 2014), TeloView (Chuang et al., 2004) and the commercially available program, Isis (MetaSystems). All pictures were converted to 16-bit individual channel monochrome images. Channels were normalized to remove background and the DAPI-positive region was manually encircled. Cy3 signals within this region are subjected to a rolling ball algorithm (mask), and halos and conjoined particles are manually eliminated/separated by the investigator. Notably, this type of editing should only be used for high-resolution images. For images captured at a lower magnification, this editing should not be used due to the small size of foci compared with the thickness of the eraser, as attempts to separate conjoined foci will result in severe signal losses. The program generates statistics on the entire region of the nucleus. Statistics returned include the intensity sum of all Cy3 telomere pixels for a given nucleus (proportional to the cell's total telomere length) and the intensity sum of all DAPI pixels for the nucleus (proportional to total cellular nuclear DNA content). Additional measurements taking into account mean intensities of both telomere signals and DAPI signals are also generated. By taking the ratio of the telomere intensity measurements to the corresponding DAPI intensity measurements, one is able to compensate for ploidy differences.

Statistical Analysis

Values are presented as mean ± SEM. Analyses were subjected to unpaired Student's t tests with Welch's correction using GraphPad Prism 6 software. Significance is indicated in the figures by $^*p \leq 0.05$ and $^{**}p \leq 0.01$. Additional information can be found in Supplemental Experimental Procedures.

SUPPLEMENTAL INFORMATION

Supplemental Information includes Supplemental Experimental Procedures, seven figures, and five tables and can be found with this article online at http://dx.doi.org/10.1016/j.stemcr.2017.08.003.

AUTHOR CONTRIBUTIONS

E.D.T. designed and performed experiments, analyzed and interpreted data, and drafted the manuscript. D.K.S. performed experiments, analyzed data, and edited the manuscript. M.T.T. carried out hMuSC isolation, analyzed data, and edited the manuscript. M.J.S. carried out mMuSC isolation and edited the manuscript. M.S.-S. performed experiments and analyzed the data. H.H and S.M. procured human biopsies. F.B.J. provided mTR^{G3} mice and edited the manuscript. A.S. designed and supervised hMuSC experiments, interpreted data, and edited the manuscript. F.M. led the conception of the MuQ-FISH method, designed and supervised the project, interpreted data, and drafted the manuscript.

ACKNOWLEDGMENTS

We thank Dr. Alan Meeker (Johns Hopkins) for sharing the software and server for telomere analysis and Nicholas Oyster for assistance with mMuSC preparation. This work was supported by Ellison Medical Foundation grant AG-NS-0843-11, the Muscular Dystrophy Association grant 200845, NIH grants UL1TR000100,

R01 AR064873, and P30 AR06130303, and SBPMDI start-up funds to A.S.; an NIH grant F31 AR065923-03 to M.T.T.; and start-up funds from the Perelman School of Medicine, a McCabe Award, and a Pilot and Feasibility grant from NIH (P30 AR069619) to F.M.

Received: January 25, 2017
Revised: August 8, 2017
Accepted: August 11, 2017
Published: September 7, 2017

REFERENCES

Aida, J. (2014). Determination of telomere length by the quantitative fluorescence in situ hybridization (Q-FISH) method. Am. J. Anal. Chem. *5*, 775–783.

Alder, J.K., Barkauskas, C.E., Limjunyawong, N., Stanley, S.E., Kembou, F., Tuder, R.M., Hogan, B.L., Mitzner, W., and Armanios, M. (2015). Telomere dysfunction causes alveolar stem cell failure. Proc. Natl. Acad. Sci. USA *112*, 5099–5104.

Armanios, M. (2013). Telomeres and age-related disease: how telomere biology informs clinical paradigms. J. Clin. Invest. *123*, 996–1002.

Armanios, M., Alder, J.K., Parry, E.M., Karim, B., Strong, M.A., and Greider, C.W. (2009). Short telomeres are sufficient to cause the degenerative defects associated with aging. Am. J. Hum. Genet. *85*, 823–832.

Baerlocher, G.M., Vulto, I., de Jong, G., and Lansdorp, P.M. (2006). Flow cytometry and FISH to measure the average length of telomeres (flow FISH). Nat. Protoc. *1*, 2365–2376.

Batista, L.F., Pech, M.F., Zhong, F.L., Nguyen, H.N., Xie, K.T., Zaug, A.J., Crary, S.M., Choi, J., Sebastiano, V., Cherry, A., et al. (2011). Telomere shortening and loss of self-renewal in dyskeratosis congenita induced pluripotent stem cells. Nature *474*, 399–402.

Blackburn, E.H., Epel, E.S., and Lin, J. (2015). Human telomere biology: a contributory and interactive factor in aging, disease risks, and protection. Science *350*, 1193–1198.

Blasco, M.A. (2007a). The epigenetic regulation of mammalian telomeres. Nat. Rev. Genet. *8*, 299–309.

Blasco, M.A. (2007b). Telomere length, stem cells and aging. Nat. Chem. Biol. *3*, 640–649.

Blasco, M.A., Lee, H.W., Hande, M.P., Samper, E., Lansdorp, P.M., DePinho, R.A., and Greider, C.W. (1997). Telomere shortening and tumor formation by mouse cells lacking telomerase RNA. Cell *91*, 25–34.

Brack, A.S., and Rando, T.A. (2012). Tissue-specific stem cells: lessons from the skeletal muscle satellite cell. Cell Stem Cell *10*, 504–514.

Calado, R.T., and Young, N.S. (2009). Telomere diseases. N. Engl. J. Med. *361*, 2353–2365.

Campbell, K.P., and Stull, J.T. (2003). Skeletal muscle basement membrane-sarcolemma-cytoskeleton interaction minireview series. J. Biol. Chem. *278*, 12599–12600.

Chang, N.C., Chevalier, F.P., and Rudnicki, M.A. (2016). Satellite cells in muscular dystrophy—lost in polarity. Trends Mol. Med. *22*, 479–496.

Chuang, T.C., Moshir, S., Garini, Y., Chuang, A.Y., Young, I.T., Vermolen, B., van den Doel, R., Mougey, V., Perrin, M., Braun, M., et al. (2004). The three-dimensional organization of telomeres in the nucleus of mammalian cells. BMC Biol. *2*, 12.

Collins, K. (2000). Mammalian telomeres and telomerase. Curr. Opin. Cell Biol. *12*, 378–383.

d'Adda di Fagagna, F., Reaper, P.M., Clay-Farrace, L., Fiegler, H., Carr, P., Von Zglinicki, T., Saretzki, G., Carter, N.P., and Jackson, S.P. (2003). A DNA damage checkpoint response in telomere-initiated senescence. Nature *426*, 194–198.

de Pauw, E.S., Verwoerd, N.P., Duinkerken, N., Willemze, R., Raap, A.K., Fibbe, W.E., and Tanke, H.J. (1998). Assessment of telomere length in hematopoietic interphase cells using in situ hybridization and digital fluorescence microscopy. Cytometry *32*, 163–169.

Decary, S., Hamida, C.B., Mouly, V., Barbet, J.P., Hentati, F., and Butler-Browne, G.S. (2000). Shorter telomeres in dystrophic muscle consistent with extensive regeneration in young children. Neuromuscul. Disord. *10*, 113–120.

Dumont, N.A., Wang, Y.X., von Maltzahn, J., Pasut, A., Bentzinger, C.F., Brun, C.E., and Rudnicki, M.A. (2015). Dystrophin expression in muscle stem cells regulates their polarity and asymmetric division. Nat. Med. *21*, 1455–1463.

Fajkus, J. (2006). Detection of telomerase activity by the TRAP assay and its variants and alternatives. Clin. Chim. Acta *371*, 25–31.

Flores, I., and Blasco, M.A. (2010). The role of telomeres and telomerase in stem cell aging. FEBS Lett. *584*, 3826–3830.

Flores, I., Canela, A., Vera, E., Tejera, A., Cotsarelis, G., and Blasco, M.A. (2008). The longest telomeres: a general signature of adult stem cell compartments. Genes Dev. *22*, 654–667.

Gardner, J.P., Kimura, M., Chai, W., Durrani, J.F., Tchakmakjian, L., Cao, X., Lu, X., Li, G., Peppas, A.P., Skurnick, J., et al. (2007). Telomere dynamics in macaques and humans. J. Gerontol. A Biol. Sci. Med. Sci. *62*, 367–374.

Guan, J.Z., Guan, W.P., Maeda, T., and Makino, N. (2013). Analysis of telomere length and subtelomeric methylation of circulating leukocytes in women with Alzheimer's disease. Aging Clin. Exp. Res. *25*, 17–23.

Hande, M.P., Samper, E., Lansdorp, P., and Blasco, M.A. (1999). Telomere length dynamics and chromosomal instability in cells derived from telomerase null mice. J. Cell Biol. *144*, 589–601.

Harley, C.B. (2005). Telomerase therapeutics for degenerative diseases. Curr. Mol. Med. *5*, 205–211.

Harley, C.B., Futcher, A.B., and Greider, C.W. (1990). Telomeres shorten during ageing of human fibroblasts. Nature *345*, 458–460.

Harley, C.B., and Villeponteau, B. (1995). Telomeres and telomerase in aging and cancer. Curr. Opin. Genet. Dev. *5*, 249–255.

Henderson, S., Allsopp, R., Spector, D., Wang, S.S., and Harley, C. (1996). In situ analysis of changes in telomere size during replicative aging and cell transformation. J. Cell Biol. *134*, 1–12.

Herbert, B., Pitts, A.E., Baker, S.I., Hamilton, S.E., Wright, W.E., Shay, J.W., and Corey, D.R. (1999). Inhibition of human telomerase in immortal human cells leads to progressive telomere shortening and cell death. Proc. Natl. Acad. Sci. USA *96*, 14276–14281.

Hovden, R., Xin, H.L., and Muller, D.A. (2011). Extended depth of field for high-resolution scanning transmission electron microscopy. Microsc. Microanal. *17*, 75–80.

Howe, B., Umrigar, A., and Tsien, F. (2014). Chromosome preparation from cultured cells. J. Vis. Exp., e50203. http://dx.doi.org/10.3791/50203.

Hultdin, M., Gronlund, E., Norrback, K., Eriksson-Lindstrom, E., Just, T., and Roos, G. (1998). Telomere analysis by fluorescence in situ hybridization and flow cytometry. Nucleic Acids Res. *26*, 3651–3656.

Joe, A.W., Yi, L., Natarajan, A., Le Grand, F., So, L., Wang, J., Rudnicki, M.A., and Rossi, F.M. (2010). Muscle injury activates resident fibro/adipogenic progenitors that facilitate myogenesis. Nat. Cell Biol. *12*, 153–163.

Kimura, M., Stone, R.C., Hunt, S.C., Skurnick, J., Lu, X., Cao, X., Harley, C.B., and Aviv, A. (2010). Measurement of telomere length by the Southern blot analysis of terminal restriction fragment lengths. Nat. Protoc. *5*, 1596–1607.

Lauzon, W., Sanchez Dardon, J., Cameron, D.W., and Badley, A.D. (2000). Flow cytometric measurement of telomere length. Cytometry *42*, 159–164.

Mann, C.J., Perdiguero, E., Kharraz, Y., Aguilar, S., Pessina, P., Serrano, A.L., and Munoz-Canoves, P. (2011). Aberrant repair and fibrosis development in skeletal muscle. Skelet. Muscle *1*, 21.

Meeker, A.K., Gage, W.R., Hicks, J.L., Simon, I., Coffman, J.R., Platz, E.A., March, G.E., and De Marzo, A.M. (2002). Telomere length assessment in human archival tissues: combined telomere fluorescence in situ hybridization and immunostaining. Am. J. Pathol. *160*, 1259–1268.

Montpetit, A.J., Alhareeri, A.A., Montpetit, M., Starkweather, A.R., Elmore, L.W., Filler, K., Mohanraj, L., Burton, C.W., Menzies, V.S., Lyon, D.E., et al. (2014). Telomere length: a review of methods for measurement. Nurs. Res. *63*, 289–299.

Morgan, J.E., and Partridge, T.A. (2003). Muscle satellite cells. Int. J. Biochem. Cell Biol. *35*, 1151–1156.

Morrison, S.J., Prowse, K.R., Ho, P., and Weissman, I.L. (1996). Telomerase activity in hematopoietic cells is associated with self-renewal potential. Immunity *5*, 207–216.

Mourkioti, F., Kustan, J., Kraft, P., Day, J.W., Zhao, M.M., Kost-Alimova, M., Protopopov, A., DePinho, R.A., Bernstein, D., Meeker, A.K., et al. (2013). Role of telomere dysfunction in cardiac failure in Duchenne muscular dystrophy. Nat. Cell Biol. *15*, 895–904.

Niida, H., Shinkai, Y., Hande, M.P., Matsumoto, T., Takehara, S., Tachibana, M., Oshimura, M., Lansdorp, P.M., and Furuichi, Y. (2000). Telomere maintenance in telomerase-deficient mouse embryonic stem cells: characterization of an amplified telomeric DNA. Mol. Cell. Biol. *20*, 4115–4127.

O'Callaghan, N.J., and Fenech, M. (2011). A quantitative PCR method for measuring absolute telomere length. Biol. Proced. Online *13*, 3.

O'Connor, M.S., Carlson, M.E., and Conboy, I.M. (2009). Differentiation rather than aging of muscle stem cells abolishes their telomerase activity. Biotechnol. Prog. *25*, 1130–1137.

O'Sullivan, J.N., Finley, J.C., Risques, R.A., Shen, W.T., Gollahon, K.A., Moskovitz, A.H., Gryaznov, S., Harley, C.B., and Rabinovitch, P.S. (2004). Telomere length assessment in tissue sections by quantitative FISH: image analysis algorithms. Cytometry A *58*, 120–131.

Ohki, R., Tsurimoto, T., and Ishikawa, F. (2001). In vitro reconstitution of the end replication problem. Mol. Cell. Biol. *21*, 5753–5766.

Ono, Y., Boldrin, L., Knopp, P., Morgan, J.E., and Zammit, P.S. (2010). Muscle satellite cells are a functionally heterogeneous population in both somite-derived and branchiomeric muscles. Dev. Biol. *337*, 29–41.

Ourliac-Garnier, I., and Londono-Vallejo, A. (2011). Telomere length analysis by quantitative fluorescent in situ hybridization (Q-FISH). Methods Mol. Biol. *735*, 21–31.

Poon, S.S., and Lansdorp, P.M. (2001). Quantitative fluorescence in situ hybridization (Q-FISH). Curr. Protoc. Cell Biol. *Chapter 18*, Unit 18 14.

Ramunas, J., Yakubov, E., Brady, J.J., Corbel, S.Y., Holbrook, C., Brandt, M., Stein, J., Santiago, J.G., Cooke, J.P., and Blau, H.M. (2015). Transient delivery of modified mRNA encoding TERT rapidly extends telomeres in human cells. FASEB J. *29*, 1930–1939.

Robin, J.D., Ludlow, A.T., Batten, K., Magdinier, F., Stadler, G., Wagner, K.R., Shay, J.W., and Wright, W.E. (2014). Telomere position effect: regulation of gene expression with progressive telomere shortening over long distances. Genes Dev. *28*, 2464–2476.

Sacco, A., Doyonnas, R., Kraft, P., Vitorovic, S., and Blau, H.M. (2008). Self-renewal and expansion of single transplanted muscle stem cells. Nature *456*, 502–506.

Sacco, A., Mourkioti, F., Tran, R., Choi, J., Llewellyn, M., Kraft, P., Shkreli, M., Delp, S., Pomerantz, J.H., Artandi, S.E., et al. (2010). Short telomeres and stem cell exhaustion model Duchenne muscular dystrophy in mdx/mTR mice. Cell *143*, 1059–1071.

Sarek, G., Marzec, P., Margalef, P., and Boulton, S.J. (2015). Molecular basis of telomere dysfunction in human genetic diseases. Nat. Struct. Mol. Biol. *22*, 867–874.

Sekulovic, S., Gylfadottir, V., Vulto, I., Gasparetto, M., Even, Y., Brookes, C., Smith, C., Eaves, C.J., Lansdorp, P.M., Rossi, F.M., et al. (2011). Prolonged self-renewal activity unmasks telomerase control of telomere homeostasis and function of mouse hematopoietic stem cells. Blood *118*, 1766–1773.

Sharpless, N.E., and DePinho, R.A. (2007). How stem cells age and why this makes us grow old. Nat. Rev. Mol. Cell Biol. *8*, 703–713.

Shay, J.W., and Wright, W.E. (2005). Senescence and immortalization: role of telomeres and telomerase. Carcinogenesis *26*, 867–874.

Shekhani, M.T., Barber, J.R., Bezerra, S.M., Heaphy, C.M., Gonzalez Roibon, N.D., Taheri, D., Reis, L.O., Guner, G., Joshu, C.E., Netto, G.J., et al. (2016). High-resolution telomere fluorescence in situ hybridization reveals intriguing anomalies in germ cell tumors. Hum. Pathol. *54*, 106–112.

Shi, X., and Garry, D.J. (2006). Muscle stem cells in development, regeneration, and disease. Genes Dev. *20*, 1692–1708.

Skalli, O., Vandekerckhove, J., and Gabbiani, G. (1987). Actin-isoform pattern as a marker of normal or pathological smooth-muscle and fibroblastic tissues. Differentiation *33*, 232–238.

Skvortsov, D.A., Zvereva, M.E., Shpanchenko, O.V., and Dontsova, O.A. (2011). Assays for detection of telomerase activity. Acta Naturae *3*, 48–68.

Thomas, M.L., and Lefrancois, L. (1988). Differential expression of the leucocyte-common antigen family. Immunol. Today *9*, 320–326.

Tierney, M.T., and Sacco, A. (2016). Satellite cell heterogeneity in skeletal muscle homeostasis. Trends Cell Biol. *26*, 434–444.

Viscardi, V., Bonetti, D., Cartagena-Lirola, H., Lucchini, G., and Longhese, M.P. (2007). MRX-dependent DNA damage response to short telomeres. Mol. Biol. Cell *18*, 3047–3058.

Wallace, G.Q., and McNally, E.M. (2009). Mechanisms of muscle degeneration, regeneration, and repair in the muscular dystrophies. Annu. Rev. Physiol. *71*, 37–57.

Wang, Y.X., and Rudnicki, M.A. (2011). Satellite cells, the engines of muscle repair. Nat. Rev. Mol. Cell Biol. *13*, 127–133.

Wong, K.K., Maser, R.S., Bachoo, R.M., Menon, J., Carrasco, D.R., Gu, Y., Alt, F.W., and DePinho, R.A. (2003). Telomere dysfunction and Atm deficiency compromises organ homeostasis and accelerates ageing. Nature *421*, 643–648.

Wu, P., Takai, H., and de Lange, T. (2012). Telomeric 3′ overhangs derive from resection by Exo1 and Apollo and fill-in by POT1b-associated CST. Cell *150*, 39–52.

Ye, J., Renault, V.M., Jamet, K., and Gilson, E. (2014). Transcriptional outcome of telomere signalling. Nat. Rev. Genet. *15*, 491–503.

Stem Cell Reports, Volume *9*

Supplemental Information

Single Stem Cell Imaging and Analysis Reveals Telomere Length Differences in Diseased Human and Mouse Skeletal Muscles

Elisia D. Tichy, David K. Sidibe, Matthew T. Tierney, Michael J. Stec, Maryam Sharifi-Sanjani, Harish Hosalkar, Scott Mubarak, F. Brad Johnson, Alessandra Sacco, and Foteini Mourkioti

Figure S1

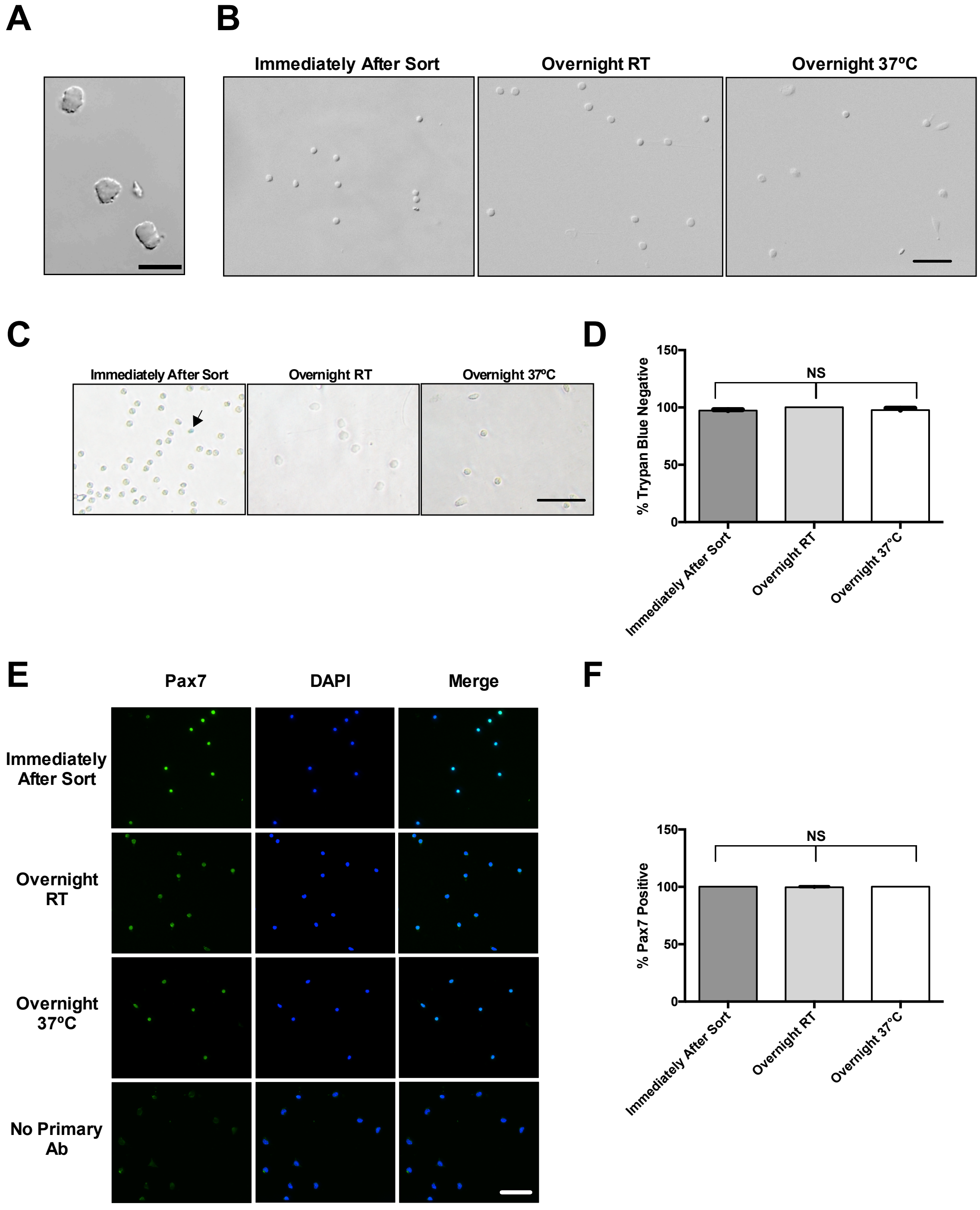

Figure S1. Telomere MuQ-FISH Processing Does Not Affect MuSC Appearance, Cell Death, or Differentiation Status. Related to Figure 1.

A) Representative Differential Interference Contrast (DIC) image of FACS-isolated mMuSCs plated on laminin-coated plates. Scale bar: 10 µm.

B) Murine MuSCs were plated on laminin-coated chamber slides and allowed to attach for 30 minutes before fixation (immediately after sort), plated and cultured overnight at 37ºC before fixation, or plated overnight at room temperature and dried before fixation. Cells were imaged by DIC microscopy. Scale bar: 100 μm.

C) MuSCs were plated as described in (B) and trypan blue was added to the culture media. In the case of the room temperature overnight group, trypan blue was added with media, to create the same final concentration. Cells were imaged by phase contrast microscopy. Scale bar: 50 μm.

D) Analysis of the percent of live cells (trypan blue negative), based on images taken in (C). n=7 mice analyzed. N>100 cells analyzed per condition. Displayed is mean ± SEM.

E) MuSCs were plated as in (B), fixed, and stained for Pax7, an undifferentiated MuSC marker. Scale bar: 100 μm.

F) Quantitation of data from (E). n=7 mice analyzed. N>50 cells analyzed per condition. Displayed is mean ± SEM.

Figure S2

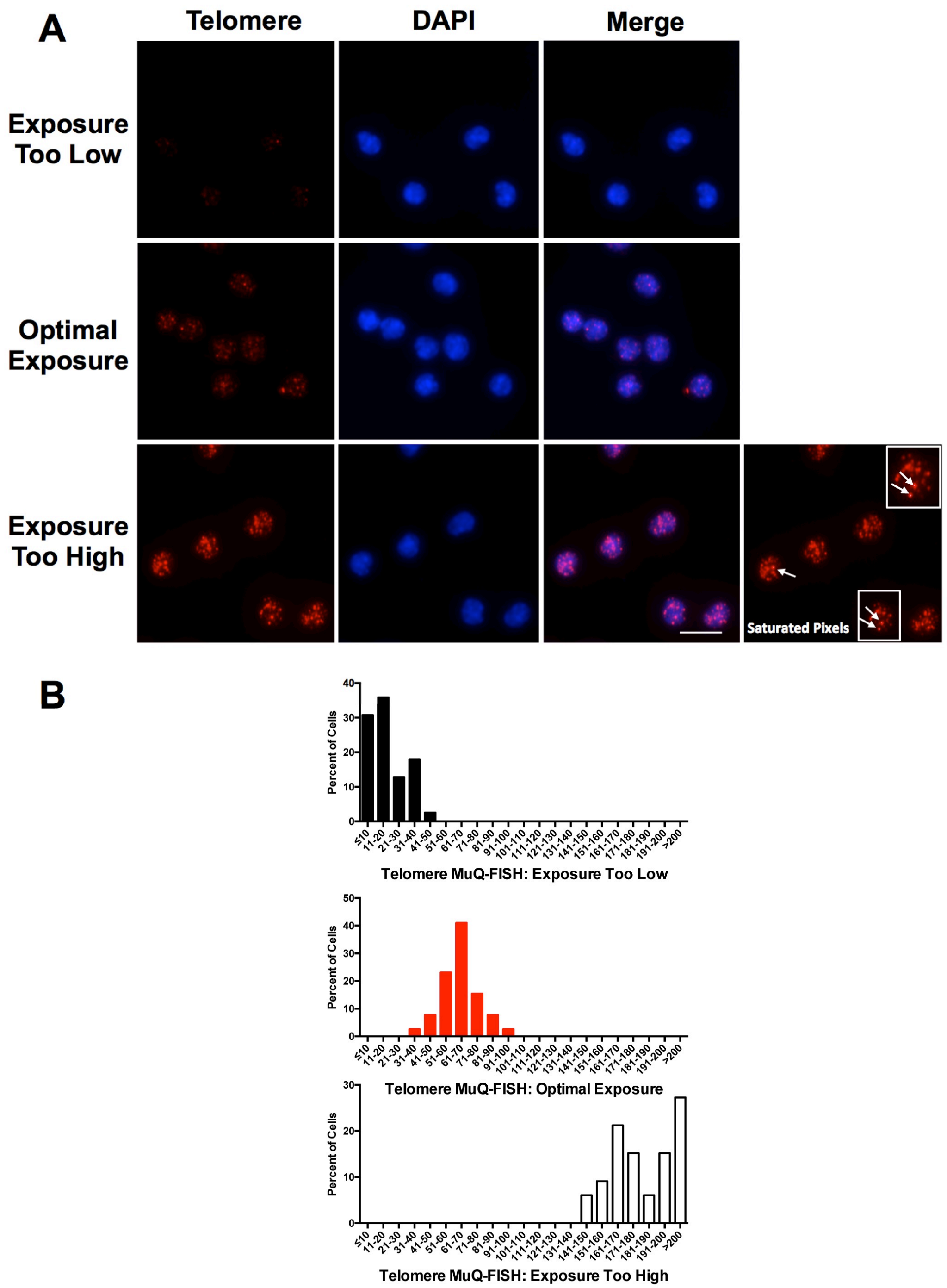

Figure S2. Determination of Optimal Exposures for MuQ-FISH. Related to Figure 1.

A) WT mMuSCs were processed for Telomere MuQ-FISH and imaged at different exposures for Cy3. It is necessary to image experimental groups to determine optimal exposure settings before collecting data. In this case, the exposure too low settings for Cy3 were 200 ms; 150 visual gain. For the optimal setting for this experiment, Cy3 was imaged at 300 ms; 150 visual gain. For the exposure too high setting where saturated

pixels can be observed (white arrows), exposure settings for Cy3 were 800 ms; 150 visual gain. DAPI images were taken at the same settings for all groups (50 ms; 206 visual gain for this experiment). Oversaturated pixels were determined using Nikon elements software. Scale bar: 5 µm. Exposure settings will vary from lots of reagents and the age of the mercury bulb in the microscopy equipment.

B) MuQ-FISH was conducted on WT MuSCs imaged with different Cy3 intensities. Data are displayed as histograms using the sum intensity information. n>30 cells/group analyzed.

Figure S3

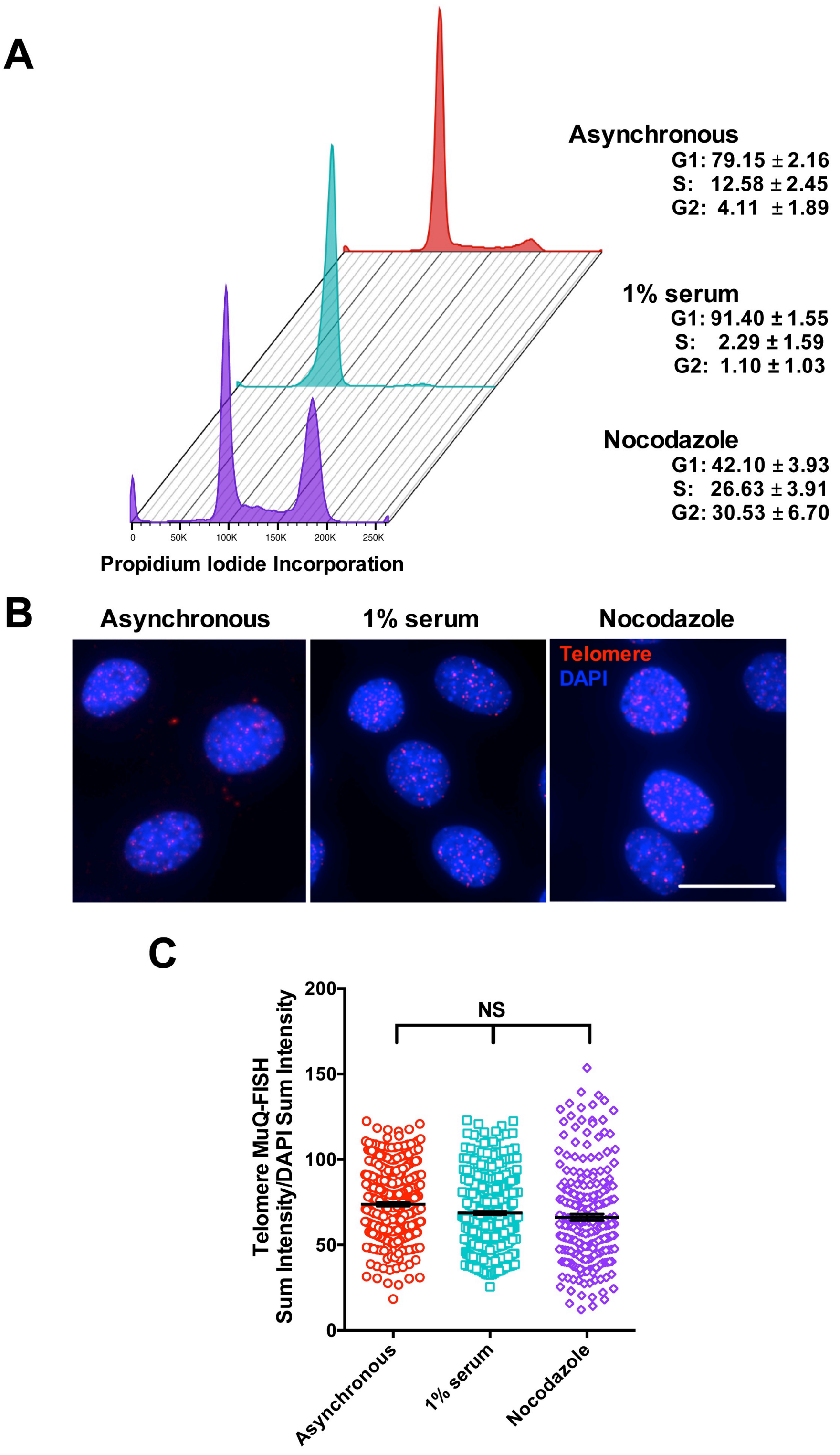

Figure S3. Cell Cycle Phase Does Not Affect Telomere Length Analysis by Telometer Related to Figure 1.

A) Undifferentiated C2C12 cells were enriched in G1 or G2 phases of the cell cycle or allowed to grow asynchronously. Cells were harvested and analyzed for cell cycle position by flow cytometry. Results were calculated from n=3 biological replicates.
B) Representative images of cells described in (A) that were processed and stained for MuQ-FISH. Cells with condensed chromatin (mitotic) were not included in the analysis. Scale bar: 25 µm.
C) Analysis of C2C12 cells by MuQ-FISH. Sum intensity method is displayed. Data presented as mean ± SEM. At least 250 cells were analyzed per group. Displayed is mean ± SEM.

Figure S4

A

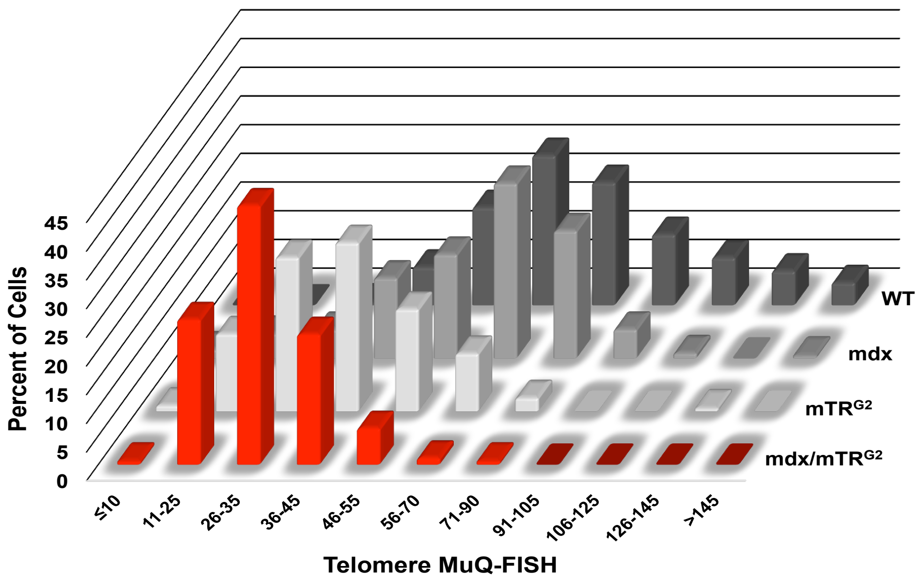

B

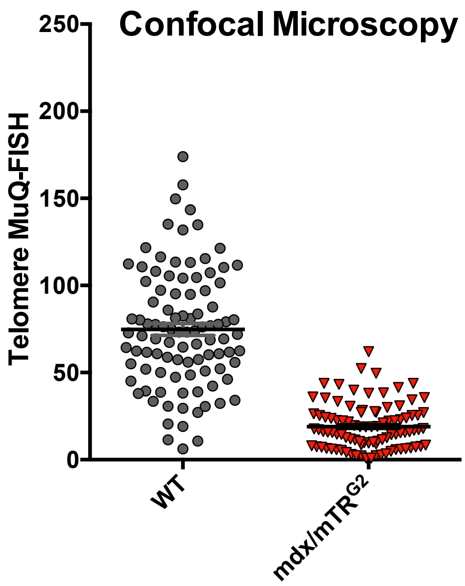

Figure S4. Telomere Shortening in Dystrophic mMuSCs. Related to Figure 4.

A) Alternative representation of the histogram in presented in Figure 4C.

B) Confocal analysis of MuQ-FISH. MuSCs from 4 WT or 4 mdx/mTR^{G2} mice were plated and processed for MuQ-FISH. Cells were imaged on a Zeiss LSM 710 confocal microscope, with 5-8 sections of 1µm per slice were taken per image. Slices were

combined in Fiji using the Z-project maximum projection algorithm. Combined Z-images of DAPI and of Cy3 were analyzed by Telometer. N>90 cells analyzed per genotype.

Figure S5

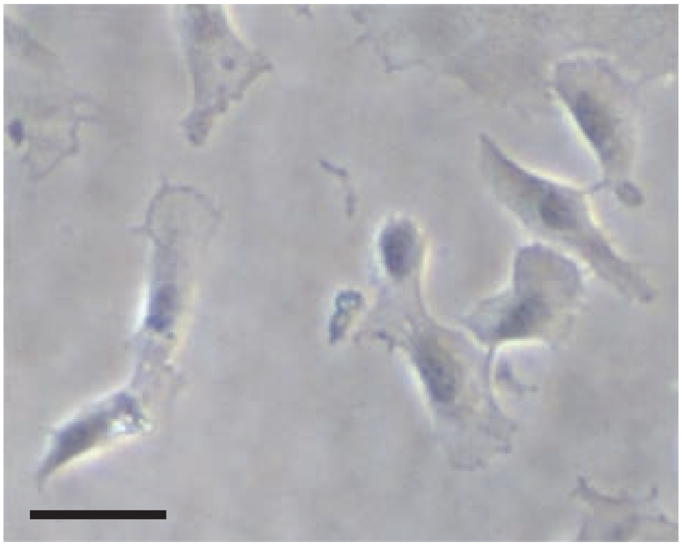

Figure S5. Morphology of Cultured hMuSCs. Related to Figure 5.
A representative image of hMuSC cells grown on collagen-coated slides, fixed, and imaged by phase contrast microscopy. Scale bar: 50 μm

Figure S6

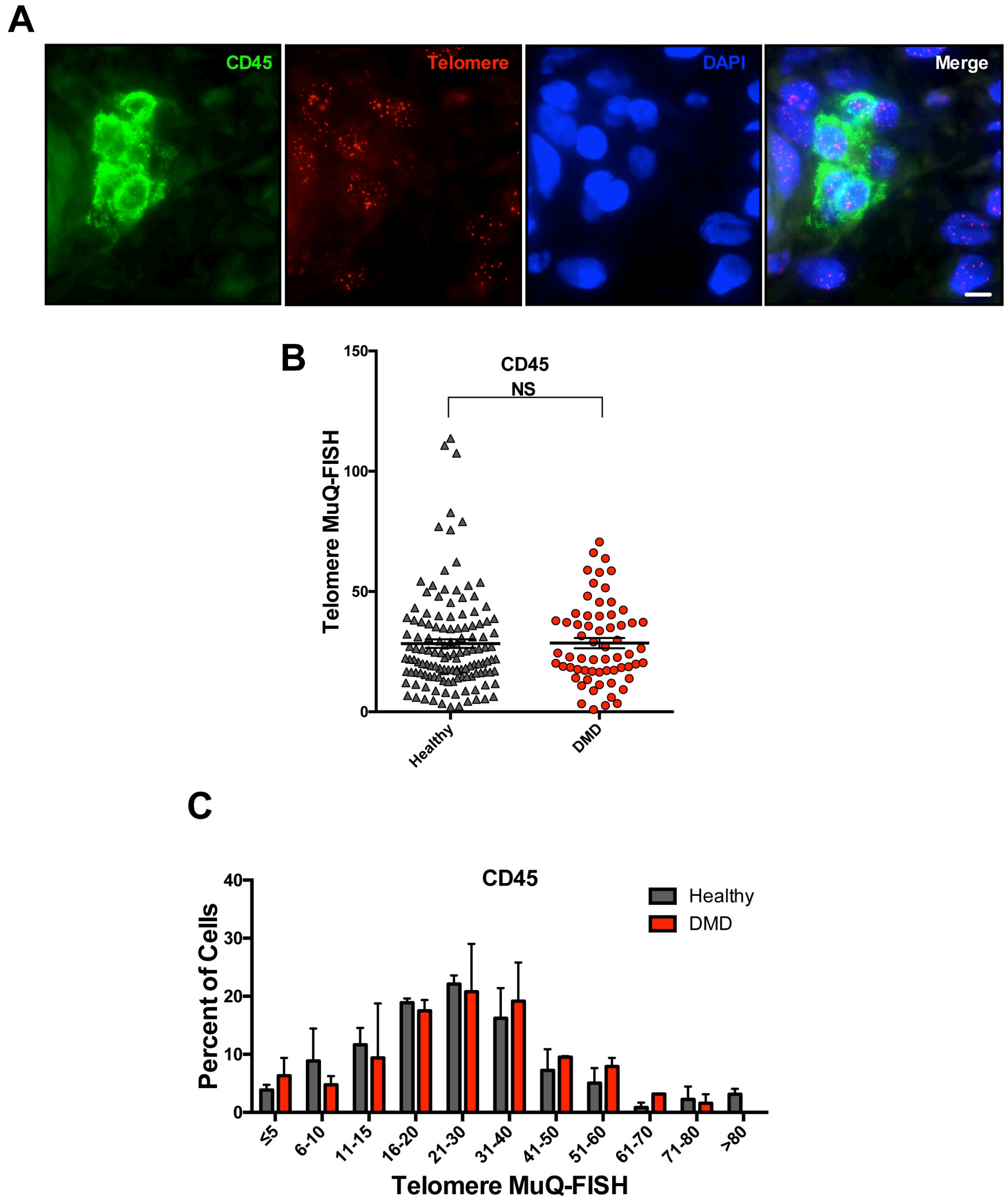

Figure S6. MuQ-FISH Staining of Leukocytes in Human Patient Samples. Related to Figure 6.

A) Skeletal muscle cryosections from human healthy or DMD-diseased patients were processed for MuQ-FISH and stained with the leukocyte marker CD45 (green). Scale bar: 10 µm.

B) Telomere length was assessed by MuQ-FISH (sum intensity) in $CD45^+$ cells in human healthy or DMD-diseased cryosections. N=3-4 patient samples per condition, n>60 $CD45^+$ cells analyzed per group. Displayed is mean ± SEM.

C) Histogram depiction of data shown in B. Displayed is mean ± SEM.

Figure S7

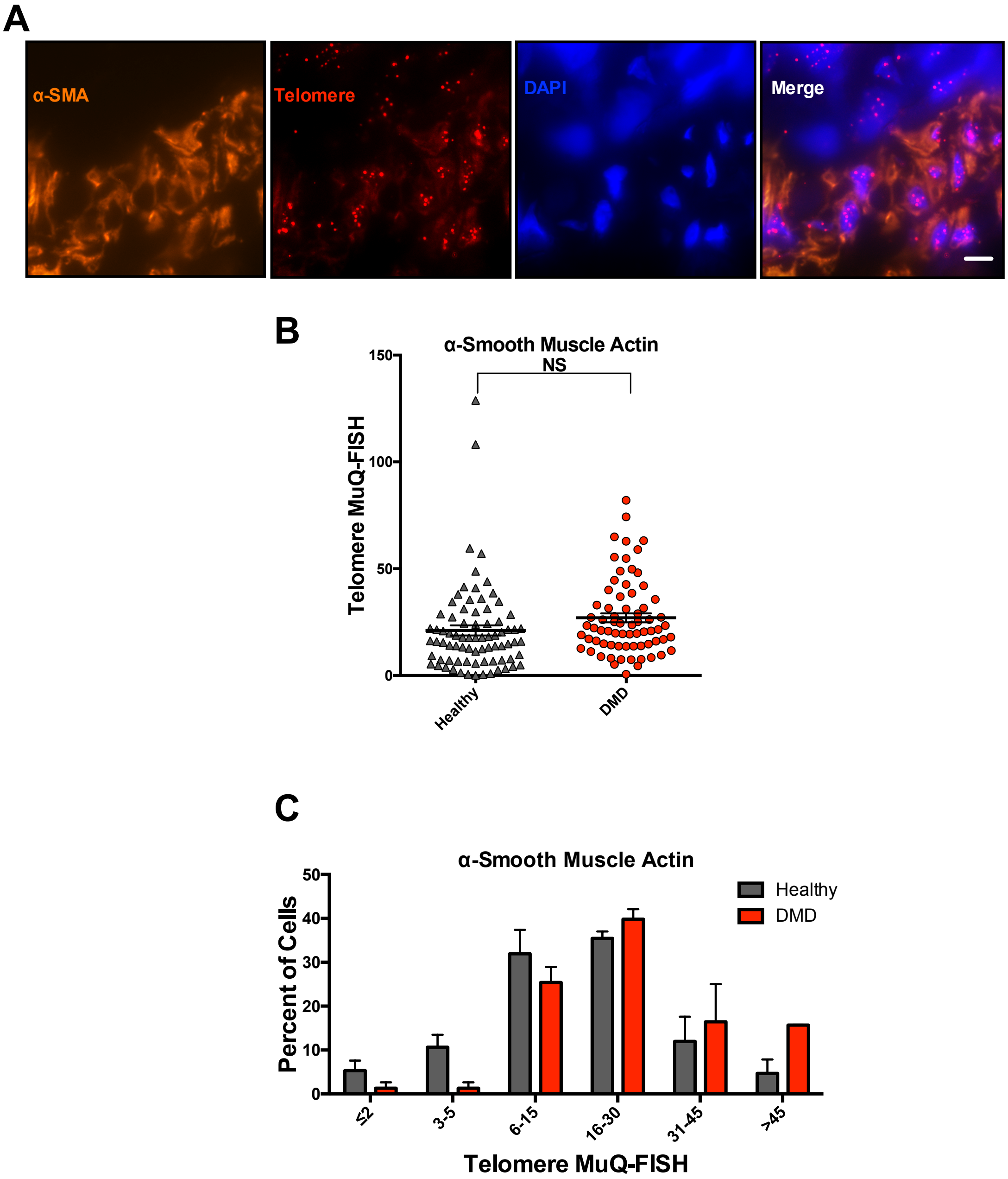

Figure S7. MuQ-FISH Staining of Smooth Muscle Cells in Human Patient Samples Related to Figure 6.

A) Skeletal muscle cryosections from human healthy or DMD-diseased patients were processed for MuQ-FISH stained with alpha smooth muscle actin (orange). Scale bar: 10 µm.

B) Telomere length was assessed by MuQ-FISH (sum intensity) in a α-SMA^+ cells in human healthy or DMD-diseased cryosections. N=3-4 patient samples per condition, n=70 α-SMA^+ cells analyzed per group. Displayed is mean ± SEM.

C) Histogram depiction of data shown in B. Displayed is mean ± SEM

Table S1. Antibodies and Staining Reagents Used in mMuSC FACS. Related to Figures 1-4.

Antigen	Host	Clone	Conjugate	Source	Dilution
**CD45**	Rat	30-F11	Biotin	BD Biosciences	1/500
**CD11b**	Rat	M1/70	Biotin	BD Biosciences	1/200
**CD31**	Rat	390	Biotin	eBioscience	1/200
**Sca1/Ly6A/E**	Rat	E13-161.7	Biotin	BD Biosciences	1/200
**Live/Dead**	N/A	N/A	7-aminoactinomycin D (7-AAD) or DAPI	Sigma-Aldrich/Thermo Fisher	1/250
**Streptavidin**	N/A	N/A	PE-Cy7 or APC-Cy7	Biolegend or BD Biosciences	1/25
**Alpha 7-Integrin**	Rat	R2F2	AF 647 or AF 488	Ablab	1/25
**CD34**	Rat	Ram34	BV421 AF 647	BD Biosciences	1/12.5

Table S2. Antibodies and Staining Reagents for hMUSC isolation. Related to Figure 5 and 6.

Antigen/Reagent	Host	Clone	Conjugate	Source	Dilution
**CD45**	Mouse	HI30	Biotin	eBioscience	1/250
**CD11b**	Mouse	ICRF44	Biotin	eBioscience	1/100
**CD31**	Mouse	WM59	Biotin	eBioscience	1/100
**Propidium Iodide**	N/A	N/A	N/A	Thermo Scientific	1/1000
**Streptavidin**	N/A	N/A	APC-Cy7	BD Biosciences	1/100
**NCAM/CD56**	Mouse	CMSSB	APC	eBioscience	1/100

Table S3: Human Subject Information. Related to Figure 5 and 6.

Patient	Group	Age (y)	Muscle	MuSC Yield
1	Healthy	13.7	Vastus lateralis	12,200
2	Healthy	14.7	Vastus lateralis	6,450
3	Healthy	14.2	Vastus lateralis	5,600
4	DMD	13.8	Gastrocnemius/soleus	2,700
5	DMD	11.0	Gastrocnemius/soleus	1,000
6	DMD	10.3	Gastrocnemius	3,400

Table S4. Fluorophores and Aria Filter Sets. Related to Figures 1-6.

Fluor	Laser	Filter
**7-AAD**	Blue	710/50
**Alexa Fluor 488**	Blue	530/30
**Alexa Fluor 647**	Red	660/20
**APC**	Red	660/20
**APC-Cy7**	Red	780/60
**BV421**	Violet	450/50
**DAPI**	Violet	450/50
**Propidium Iodide**	Blue	585/42
**PE-Cy7**	Green	780/60

Table S5. Formulas Used for MuQ-FISH Analysis. Related to Figures 2, 3, 4, 6.

**Sum Intensity**	$\frac{\textbf{Sum Intensity of Telomere Signal}}{\textbf{Sum Intensity of DAPI Signal}}$
**Mean Intensity**	$\frac{\textbf{Mean Intensity of Telomere Signal}}{\textbf{Sum Intensity of DAPI Signal}}$

Note: After ratio calculations, all values were multiplied by a conversion factor to generate values greater than 1.

Extended Experimental Procedures

Mice

Wildtype C57Bl/6J mice were purchased from Jackson Labs (stock #000664). Other mice used in the study included mdx (Jackson Labs stock #002378), mTR^{G1} (generation 1 mTR knockout, Jackson Labs stock #004132, derived from a heterozygous cross) mTR^{G2} (generation 2; generated through in-house by breeding homozygous Jackson Labs stock #004132), mTR^{G3} (generation 3) and mdx/mTR^{G2} (generation 2), the latter of which were generated through an in-house breeding, as described previously (Mourkioti et al., 2013; Sacco et al., 2010).

Murine Muscle Digestion and Cell Staining

Mice were sacrificed, and the tibialis anterior, quadriceps and gastrocnemius muscles were dissected from both hind legs. Muscle was finely minced and placed in a gentleMACS C tube (Miltenyi Biotec) containing 0.15% collagenase in 10 mL DMEM. Tubes were loaded into a MACS Dissociator (Miltenyi Biotec) and the manufacturer's spleen-02 program was run twice. Tubes were incubated in a humidified 37ºC/5% CO_2 incubator for 30 min, subjected to the spleen-02 program again, and incubated at 37ºC for 1 hr. Seventy five microliters of 2% collagenase (Sigma-Aldrich) and 75 µL of 4.8 U/mL dispase (Roche) was added, and tubes were vortexed at maximum speed prior to a 30-min incubation at 37ºC. Cells were passed through a 21-gauge needle until all muscle was broken apart. The remaining cell slurry was filtered through a 40 µM cell strainer that was prewet with 10 mL of cold myoblast media [DMEM:F12; 15% FBS, 1X anti-anti (Amphotericin B, Penicillin, Streptomycin); all from Gibco]; the strainer was rinsed with an additional 10 mL of cold myoblast media, and cells were pelleted at 300x*g*/4ºC. Cells were incubated with 1mL 1X red cell lysis buffer (eBioscience) for 5 min at room temperature and 9 mL cold FACS buffer (2.5% goat serum, 2 mM EDTA, pH 8.0 in 1X PBS) was added. Cells were spun and resuspended in 1mL of FACS buffer containing antibodies raised against antigens CD45, CD31, CD11b, and Sca1 (see Table S1) prior to an incubation on ice for 45 min. Cells were centrifuged and resuspended in 100 µL of FACS buffer containing antibodies CD34, and α7-integrin, as well as streptavidin-PE-Cy7 (Table S1). Cells were incubated in the dark for 90 min on ice, with agitation occurring every 30 min. FACS buffer was added up to 1 mL final volume, and cells pelleted and resuspended in FACS buffer, and the viability dye 7-aminoactinomycin D (7-AAD; Sigma-Aldrich) was added (final concentration 4 µg/mL). Cells were placed in flow cytometry tubes with cell strainers (BD Biosciences) before collection by FACS. In some cases, the antibody fluorescent conjugate choice was altered (see Table S1).

Human Muscle Biopsy Digestion and Cell Staining

Human biopsies were enzymatically dissociated (0.2% collagenase and 0.02 units/mL dispase, Sigma-Aldrich) for 45 min at 37ºC, minced under a dissection microscope and incubated for an additional 45 min. The resulting cell suspension was filtered through a 70 μm nylon filter and incubated with the following biotinylated antibodies: CD45, CD11b and CD31 (eBioscience; See Table S2). Samples were washed and incubated with anti-NCAM/CD56 (eBioscience) and Streptavidin APC-Cy7 (BD Biosciences). Dead cells were excluded by Propidium Iodide incorporation (Thermo Scientific) before fractionation of the $CD45^{-}CD11b^{-}CD31^{-}NCAM/CD56^{+}$ population by flow cytometry (BD FACS Aria II). Purity checks were performed by re-sorting an aliquot of each sorted population. Patient sample metrics can be found in Table S3.

Fluorescence-Activated Cell Sorting (FACS)

Both mMuSCs and hMuSCs were sorted using a BD Aria II. Information about lasers and filter sets can be found in Table S4. mMuSCs were sorted into 1.5 mL microcentrifuge tubes containing 500 μL of cold myoblast media [DMEM/Ham's F12; 15% FBS; 1X anti-anti (Fisher Scientific)]. The collection tubes were maintained at 4ºC during the sort process using a circulating water system. Data was collected from 20,000 total events, unless otherwise noted, and analyzed using FlowJo 10.1 software.

hMuSC culture

hMuSCs were plated on tissue culture plates coated with collagen (BD Biosciences) and maintained in growth media composed of Ham's F-10 (Gibco) or DMEM/Ham's F12 (Gibco) and 15% FBS (Omega Scientific). hMuSCs were expanded and passaged by dissociation with Accumax (Millipore). Healthy lines were used at passages 7-10 for this study, whereas DMD lines were used at passages 5-7.

Trypan Blue Staining

To determine viability, MuSCs were plated in laminin-coated 16 well chamber slides in 100 μL myoblast media. Cells were allowed to attach for 30 minutes in a 37ºC/5% CO_2 incubator. Trypan blue (Corning; 1/10 dilution of a 0.4% w/v solution in PBS) was added to the media and cells were imaged by phase microscopy (immediately after sort). MuSCs were also plated on chamber slides and left overnight in the incubator or placed at room temperature without the lid to dry. The following day, trypan blue was added to the culture media for cells in the incubator, or mixed up 1/10 with myoblast media and added to the dried room temperature wells. Cells were also imaged by phase microscopy.

Pax7 Staining

Mouse MuSCs were plated on laminin-coated 4-well chamber slides in a manner identical to the trypan blue staining procedure above. Cells were fixed with 4% paraformaldehyde/PBS for 20 min and washed once with PBS. Cells were permeabilized with 0.5% triton X-100/PBS for 30 min, washed twice with PBS, and blocked for 1 hr at room temperature in 3% BSA/PBS. Cells were stained for Pax7 (santa cruz; clone PAX7; 1/50 in 3% BSA/PBS) overnight at 4ºC, washed with PBS, and stained with Alexa Fluor 488-conjugated goat anti-mouse IgG (Life Technologies; 1/500 in 3% BSA/PBS) at room temperature for 1 hr. Cells were washed, chambers removed, and coverslips were mounted with fluoromount G plus DAPI (SouthernBiotech).

Supplemental Imaging of mMuSCs and hMuSCs.

Isolated mMuSCs were plated on laminin-coated slides. hMuSCs were grown on collagen-coated chamber slides. Both cell types were fixed with 4% paraformaldehyde/PBS for 10 min, and coverslips were mounted with Fluoromount G. mMuSCs were imaged by differential interference contrast microscopy using a Leica SP8 confocal microscope with a Nikon Plan Apo CS2 63x/1.40 oil objective. hMuSCs were imaged with a Nikon TE300 inverted CCD SPOT RT camera using a 10X air objective. For DIC imaging of mMuSCs plated and harvested at different time points after sorting, mMuSCs were plated as described in trypan blue staining above, fixed in 4% paraformaldehyde in PBS, and coverslips were mounted with fluoromount G (SouthernBiotech) before imaging.

Human Muscle Cryosection MuQ-FISH

Cryosections (10 μm slices) from human biopsies from control (healthy) or DMD-diseased individuals were fixed with 4% paraformaldehyde/PBS and permeabilized in 1% tween 20/water. Antigen retrieval with sodium citrate buffer was conducted in a steamer for 30 min, and the slides were allowed to cool for 20 min. MuQ-FISH staining proceeded in the same fashion as for MuSCs, with the exception of washes. After the typical 6 washes with SSC buffers, two additional washes of 5 min each were completed with 0.25X SSC/0.1% tween 20 at 55ºC. After rinsing with PBS, slides were blocked with protein block (Dako) for 1 hr at room temperature. Antibodies against human CD45 (Dako, clone LCA; 1/250) or alpha smooth muscle actin (Abcam; clone E184, 1/200) were diluted in antibody dilutent (Dako) and incubated in a dark, humidified chamber overnight at 4ºC. Slides were washed the following day with PBS and incubated with secondary antibodies (Alexa Fluor 647-conjugated goat anti-rabbit IgG or Alexa Fluor 488-conjugated goat anti-mouse IgG; both at 1/250) in antibody dilutent for 1 hour at room temperature in the dark. After washing with PBS, coverslips were mounted with fluoromount G. MuQ-FISH imaging and analysis was completed on a single section, using methodology described previously in this manuscript.

C2C12 Cell Synchronization, Cell Cycle Analysis, and MuQ-FISH

The mouse myoblast cell line C2C12 was grown in DMEM supplemented with 10% FBS, 1X glutamax, and 1X anti-anti. For synchronization experiments, C2C12 cells were sparsely plated in 60mm dishes (for cell cycle) or 4 well chamber slides (for MuQ-FISH). The following day, cells were washed twice with PBS, and grown in either normal growth media (asynchronous), in DMEM containing only 1% serum for 36 hr (enriched in G1), or grown in growth media supplemented with 200 ng/mL nocodazole for 12 hr (enriched in G2/M), as described (Tintignac et al., 2004). Following incubations, cells were prepared for cell cycle analysis as described (Myer et al., 2011). Briefly, cells were trypsinized, washed with PBS, and fixed with cold 70% ethanol for 15 min. Cells were pelleted and resuspended in PBS, and treated with 40 µg/mL of boiled RNAse A. Cells were incubated at 37ºC for 20 min, and 10 µg/mL of Propidium iodide was added. Cells were analyzed on an LSR II flow cytometer. Figures were prepared with FLOWJO v.10.1. n=3 biological replicates of 20,000 events/sample. For MuQ-FISH, cells were imaged with a Nikon Plan Apo 40X/0.95 objective. The sum intensity values were used to generate figures.

Supplemental References

Mourkioti, F., Kustan, J., Kraft, P., Day, J.W., Zhao, M.M., Kost-Alimova, M., Protopopov, A., DePinho, R.A., Bernstein, D., Meeker, A.K., *et al.* (2013). Role of telomere dysfunction in cardiac failure in Duchenne muscular dystrophy. Nat Cell Biol *15*, 895-904.

Myer, D.L., Robbins, S.B., Yin, M., Boivin, G.P., Liu, Y., Greis, K.D., Bhassi, el M., and Stambrook, P.J. Absence of polo-like kinase 3 in mice stabilizes Cdc25A after DNA damage but is not sufficient to produce tumors. Mutat Res *714*, 1-10.

Sacco, A., Mourkioti, F., Tran, R., Choi, J., Llewellyn, M., Kraft, P., Shkreli, M., Delp, S., Pomerantz, J.H., Artandi, S.E., *et al.* (2010). Short telomeres and stem cell exhaustion model Duchenne muscular dystrophy in mdx/mTR mice. Cell *143*, 1059-1071.

Tintignac, L.A.J., Sirri, V., Leibovitch, M.P., Lécluse, Y., Castedo, M., Metivier, D., Kroemer, G., and Leibovitch, S.A. (2004). Mutant MyoD lacking Cdc2 phosphorylation sites delays M-phase entry. Mol Cell Biol *24*, 1809-1821.
